# Topical Application of the PI3Kβ-Selective Small Molecule Inhibitor TGX-221 Is an Effective Treatment Option for Experimental Epidermolysis Bullosa Acquisita

**DOI:** 10.3389/fmed.2021.713312

**Published:** 2021-09-07

**Authors:** Hannah Zillikens, Anika Kasprick, Colin Osterloh, Natalie Gross, Michael Radziewitz, Cindy Hass, Veronika Hartmann, Martina Behnen-Härer, Nancy Ernst, Katharina Boch, Gestur Vidarsson, Remco Visser, Tamás Laskay, Xinhua Yu, Frank Petersen, Ralf J. Ludwig, Katja Bieber

**Affiliations:** ^1^Lübeck Institute of Experimental Dermatology and Center for Research on Inflammation of the Skin, University of Lübeck, Lübeck, Germany; ^2^Priority Area Asthma and Allergy, Research Center Borstel, Airway Research Center North, German Center for Lung Research, Borstel, Germany; ^3^Department for Infectious Diseases and Microbiology, University of Lübeck, Lübeck, Germany; ^4^Sanquin Research and Landsteiner Laboratory, Amsterdam, Netherlands

**Keywords:** neutrophils, bullous skin diseases, signaling, PI3K, immune-complex induced autoimmunity

## Abstract

Class I phosphoinositide 3-kinases (PI3K) have been implemented in pathogenesis of experimental epidermolysis bullosa acquisita (EBA), an autoimmune skin disease caused by type VII collagen (COL7) autoantibodies. Mechanistically, inhibition of specific PI3K isoforms, namely PI3Kβ or PI3Kδ, impaired immune complex (IC)-induced neutrophil activation, a key prerequisite for EBA pathogenesis. Data unrelated to EBA showed that neutrophil activation is also modulated by PI3Kα and γ, but their impact on the EBA has, so far, remained elusive. To address this and to identify potential therapeutic targets, we evaluated the impact of a panel of PI3K isoform-selective inhibitors (PI3Ki) on neutrophil function *in vitro*, and in pre-clinical EBA mouse models. We document that distinctive, and EBA pathogenesis-related activation-induced neutrophil *in vitro* functions depend on distinctive PI3K isoforms. When mice were treated with the different PI3Ki, selective blockade of PI3Kα (alpelisib), PI3Kγ (AS-604850), or PI3Kβ (TGX-221) impaired clinical disease manifestation. When applied topically, only TGX-221 impaired induction of experimental EBA. Ultimately, multiplex kinase activity profiling in the presence of disease-modifying PI3Ki identified unique signatures of different PI3K isoform-selective inhibitors on the kinome of IC-activated human neutrophils. Collectively, we here identify topical PI3Kβ inhibition as a potential therapeutic target for the treatment of EBA.

## Introduction

Class I phosphoinositide 3-kinases (PI3Ks) are heterodimers consisting of one regulatory and one homologous p110 catalytic subunit. The p110α and p110β isoforms are ubiquitously expressed and mice lacking p110α or p110β die during embryonic development (1, 2). The p110δ subunit is primarily found in leukocytes (3, 4), and has essential roles in development and function of lymphocytes, mast cells and possibly neutrophils (5–7). The p110γ subunit is highly expressed in the immune system but also in other tissues, including heart and central nervous system (8, 9). Regarding its role in immune homeostasis, aberrant PI3K signaling may lead to hematologic malignancies or chronic inflammatory diseases (10, 11).

In pemphigoid diseases, a group of autoimmune skin diseases, neutrophils are recruited to the sites of autoantibody deposits and are activated by binding to the tissue-bound immune complexes (ICs) (12). Binding to ICs is facilitated by FcγR expressed by neutrophils. Signaling downstream of activating FcγR is initiated by receptor clustering, leading to activation of Src family kinases and subsequently SYK. Next, downstream kinases, including PI3K, are activated (13–15). Neutrophils express all 4 class I PI3K isoforms (16, 17). Of these, PI3Kβ/γ/δ, but not p110α, have been implicated to regulate neutrophil activation. More specifically, PI3Kβ (16) and PI3Kδ (18) were shown to be crucial for IC-induced neutrophil activation, specifically the release of reactive oxygen species (ROS). PI3Kγ controls ROS release from fMLP-stimulated human and murine neutrophils (19), as well as neutrophil migration toward GM-CSF (17). PI3Kδ inhibition also leads to selective impairment of neutrophil functions, including IC-, C5a-, and fMLP-induced ROS release, while no impact on PMA-induced ROS release is observed. Furthermore, IL-8- or fMLP-induced migration are impaired by PI3Kδ inhibition, while C5a-induced migration remains unaffected (18). Furthermore, TNF- and LPS-induced cytokine release from neutrophils (19, 20) and extravasation (7) are controlled by PI3Kδ.

Epidermolysis bullosa acquisita (EBA) is a chronic autoimmune pemphigoid-like disease characterized and caused by autoantibodies targeting type VII collagen (COL7) (21, 22). Two distinct clinical EBA manifestations have been described: (i) mechano-bullous EBA and (ii) inflammatory EBA. Overall, EBA is notoriously difficult to treat—combined immunosuppressive treatment induces remissions after 9 months (23–25). Therefore, the implementation of novel therapeutic strategies would be of great benefit. Insights from model systems of the disease that reflect the inflammatory variant of EBA (26) showed that IC activation of neutrophils is a key prerequisite to induce tissue pathology in EBA (21). In line with this notion, inhibition of class I PI3K has been shown to dampen clinical disease manifestation in pre-clinical EBA models. More specifically, PI3Kβ deficient mice are almost completely protected from induction of experimental EBA by transfer of COL7 antibodies (16), and pharmacological PI3Kδ inhibition has therapeutic effects in immunization-induced EBA (18). The impact of the PI3Kα and γ-isoforms on EBA has, however remained elusive. Systemic blockade of the PI3K signaling pathway is associated with severe adverse events, i.e., neutropenia and pulmonary infections (27). Thus, for the treatment of EBA, topical application of PI3K inhibitors (PI3Ki) would be ideal in terms of efficacy and safety. To address these questions, we here used a panel of 7 PI3Ki with selectivity profiles covering all PI3K isoforms in experimental models of EBA.

Based on the current understanding of autoantibody-induced tissue pathology in the inflammatory variant of EBA (12, 21), where neutrophils migrate form the blood into the skin, form an immunological synapse by binding to the immune complexes located at the dermal-epidermal junction, and ultimately release ROS, we evaluated the impact of the PI3Ki on (i) IL-8-induced neutrophil migration, (ii) IC-induced neutrophil spreading, and (iii) IC-induced ROS release form neutrophils. For pre-clinical translation, the impact of systemic and topical PI3Ki treatment was evaluated in antibody transfer-induced EBA.

## Materials and Methods

### Studies With Human Biomaterial

For isolation of PMNs and peripheral blood mononuclear cells (PBMCs), normal human blood was obtained. Healthy controls gave their written informed consent prior to study participation. All of the experiments using human samples were approved by the local ethics committee (University of Lübeck, Lübeck, Germany, AZ 09-140 and AZ 20-341) and were performed in accordance with the Declaration of Helsinki.

### Mice

C57BL/6J mice (Charles River, Sulzfeld, Germany) were bred in a specific pathogen free environment and provided standard mouse chow and acidified drinking water *ad libitum*. Gender-matched mice were used for experimental EBA models at the age of 8–10 weeks. Animal experiments were approved by local authorities of the Animal Care and Use Committee (Kiel, Germany) and performed by certified personnel [AZ122.5(108/08-15)] following the ARIVE guidelines.

### Chemicals

If not otherwise stated, all other chemicals were applied from Merck or Sigma. All PI3K blocker were supplied by Selleckchem (Houston, Tx, USA). A list of used drugs including IC_50_ values and concentration of oral administration is prepared as Supplementary Table 1. For *in vitro* assays, 10 mM were dissolved in DMSO and further diluted in RPMI media. Final concentration for all *in vitro* assays were 1,000, 100, 10, 1, and 0.1 nM. For all *in vitro* assays, the final DMSO concentration was adjusted to 0.01%. All *in vitro* data were recorded in technical duplicates, and the mean of the results was normalized to the value obtained from stimulated cells without PI3K inhibition.

For *in vivo* experiments (oral administration), PI3K inhibitors were dissolved in 10% DMSO/0.8% methylcellulose/0.1% Tween-80. For topical administration to the ear 100 μl 0.9 mg/ml PI3K inhibitors in DMSO: Acetone, 1: 25 were administered daily by dropping.

### Human Polymorphonuclear (PMN) Purification

For the PamGene analysis, FACS, NET formation, cytokine release, and ROS release assays, human PMNs were isolated from whole blood samples using a PolymorphPrep™ (Progen, Heidelberg, Germany) gradient according to the manufacturer's instructions. The purity of the PMNs was evaluated by flow cytometry (MACSQuant^®^ Analyzer 10, Miltenyi) using fluorescent staining with anti-human CD14 (clone HCD14, Biolegend) and anti-human CD15 antibodies (clone VIMC6, Miltenyi). For all experiments, the purity of PMNs was >85%.

For chemotaxis assay, PMNs were isolated from normal human blood using Ficoll™ (28). In detail, blood was diluted 1:3 in HES/PBS (1:1) and allowed to sediment for 45 min. Supernatant (20 ml) was slowly added to 8 ml of Ficoll™ and centrifuged at 850 × g for 25 min (RT) without braking. The sediment (containing granulocytes) was erythrocyte lysed with Aq. dest. for 45 s, and the reaction was stopped with the same volume of 2xPBS After washing, the cells were diluted to 4 × 10^6^/ml in RPMI 1640 (without phenol red) containing 0.5% bovine serum albumin (BSA).

For spreading assay, human blood is mixed 1: 2 with 1% Polyvinylalcohol at RT, and left at RT for 30–45 min and slowly layered onto Pancoll (PAN biotech, Aidenbach, Germany). The tube is centrifuged for 20min without brake at 850 × g at RT. The supernatant is discarded and the PMNs are transferred into a fresh 50 ml tube by prior resuspension and erythrocyte lysis with 1 ml of distilled water for a total of 45 s. The lysis is stopped with 2xPBS and the tube is filled up with 1xPBS (without Ca/Mg). After centrifugation, the cell pellet is washed and then resuspended with RPMI 1640 (without phenol red) containing mit Glutamin, 2 g/L NaHCO3,1% FCS, 2 g/L Glucose 25 mM HEPES at a concentration of 2 × 10^6^ cells/ml.

### IC-Induced ROS Release From PMNs

A LumiTrack™ high binding 96-well-plate (Thermo Fisher Scientific, Waltham, MA, USA) was coated with immunocomplexes (ICs) consisting of human COL7E-F antigen at a final concentration of 2.5 μg/ml and anti-human COL7 IgG1 antibody at a final concentration of 1.8 μg/ml, as described previously (29). A total of 2 × 10^5^ cells were added per well in the presence/absence of PI3K inhibitors at the indicated concentrations. As controls, antigens or antibodies alone were added to the wells. Just before the measurement was taken, luminol was added to the wells, and the chemiluminescence resulting from the ROS production was measured immediately in a luminescence reader (Perkin Elmer GloMax). The ROS release was measured for 1 s per well, sixty-six times for a period of ~2 h at a constant temperature of 37°C (29).

### Neutrophil Adhesion and Spreading on IC

A 96-well E-plate (ACEA bioscience, Bremen, Germany) was coated with 0.5 μg/well mCOL7 and incubated overnight at 4°C. The E-plate is washed with PBS/1%BSA/0.05% Tween20 and dried for 2 h at 37°C with 5% CO_2_. Thereafter, the plate was incubated with 1.8 μg/ml anti mCOL7-IgG for 1 h at 37°C to perform immobilized ICs. The plate is then washed again and incubated with the cell suspension (2 × 10^6^/ml) w/o PI3K inhibitors. IC-stimulated with vehicle cells serve as positive control and the negative control consists of unstimulated cells, antigen or antibodies and cells. Adhesion was measured for 2 h. Neutrophil adhesion was monitored by phase contrast microscopy quantified by real-time impedance measurement by using the xCELLigence system (Roche, Penzberg, Germany) (30). The electrical impedance reflects the surface area covered by cells, and is therefore associated with the immune complex mediated neutrophil adhesion. It is expressed as cell index in arbitrary units. As microscopic control, cells were stimulated in addition with ICs consisting of human COL7E-F antigen at a final concentration of 2.5 μg/ml and anti-human COL7 IgG1 antibody at a final concentration of 1.8 μg/ml for 2 h at 37°C and treated w/o 10 μM of the respective PI3K inhibitors.

### IC-Induced Stimulation of PMNs and Determination of Cytokine Concentrations and Toxicity

A high binding 96-well-plate (Thermo Fisher Scientific) was coated with ICs consisting of human COL7E-F antigen at a final concentration of 2.5 μg/ml and anti-human COL7 IgG1 antibody at a final concentration of 1.8 μg/ml, as described previously (29). A total of 2 × 10^5^ cells were added per well w/o PI3K inhibitors at the indicated concentrations. As controls, antigens or antibodies alone were added to the wells. Cells were incubated for 2 h at 37°C in an atmosphere containing 5% CO_2_. The supernatant was analyzed by Legendplex (Biolegend, San Diego, USA) for TNF and IL-6 among others (for a list see Supplementary Table 2) following the manufacturer's protocol. For FACS analysis, single cells were stained for the following surface markers using standard FACS procedures: anti-CD15 (clone VIMC6) and anti-CD45 (clone H130) both from Biolegend or Miltenyi. For live/dead staining, Annexin V and PI were used in accordance with the manufacturer's protocol. Cells were first gated for scatter (SSC-A/FSC-A) and singlets (FSC-H/FSC-A). The CD45+/CD15+ gates were further analyzed for double-positive staining of PI and Annexin V for the measurement of toxicity/survival (CD15^pos^CD45^pos^PI^neg^Annexin V^neg^ cells are identified as live cells). Measurements were performed on a Miltenyi MacsQuant10, and data were analyzed with the MACSQuantify™ Software (Version 2.11).

### IL-8-Induced Neutrophil Chemotaxis

Chemotaxis was measured using a 48-well Boyden chamber (NeuroProbe Inc., Cabin John, MD) as described previously (31). The chamber was pretreated as described in the manufacturer's protocol. The bottom wells were blocked for 1 h with blocking buffer (1% BSA in 0.1 M NaHCO_3_/0.1 M Na_2_CO_3_, pH = 9) at 37°C. The buffer was carefully removed, and the chamber was dried at 37°C. The bottom wells were filled with 30 μl of 6–12 nmol/L human IL-8 (Peprotech, Hamburg, Germany) in 0.5% BSA/PBS (with Ca/Mg). The chambers were covered with a polycarbonate membrane (pore size, 3 μm; Costar Nucleopore GmbH, Tübingen, Germany) and incubated for 30min at 37°C. Finally, the top wells received 50–80 μl of the isolated PMNs containing the appropriate concentrations of PI3K inhibitors and were incubated for 1 h at 37°C in an atmosphere containing 5% CO_2_.

The assay was stopped by replacing the cell suspension in the upper well for another 5 min to completely detach migrated cells from the bottom side of the filters. Then, filters were removed, and the migrated cells were transferred from the bottom wells to a microtiter plate. Residual cells in the bottom wells were lysed by adding 25 μl of 0.2% hexadecyltrimethylammonium bromide/0.1% BSA in PBS, and were combined with the cells transferred to the microtiter plate; cell lysis was continued for 15min. Then, 50 μl of TMB solution (Thermo Fisher Scientific) was added until a color reaction was visible, and the enzymatic reaction was stopped by adding 50 μl 1 M H_2_SO_4_. The color reaction was determined at 450 nm in a microplate reader. The number of migrated cells was calculated from a standard of lysed cells run in parallel.

### Measurement of C5a and IL-8 Release From Keratinocytes

Affinity purified antibodies against hCOL7 E-F (EBA) were isolated from patient immunapheresis material as described (32, 33). Briefly, antigen stock solutions were prediluted to a concentration of 1.5 mg/ml in stock solvent (PBS), then further diluted 1:3 in 0.1 M Borat buffer (pH 9.5) (final volume 12 ml). Antigens were subsequently coupled to NHS-Sepharose Fast Flow^4^ columns (GE Healthcare, Chicago, USA) following manufacturer's instructions. Patient immunapheresis material was added to the corresponding column and incubated for 30min at 4°C. Afterwards, the column was washed with PBS until OD_280_ of the flow through was lower than 0.01, and the bound antibody was eluted in glycine buffer (pH 2.8). The eluate was adjusted to pH 7 using 1M tris base solution (pH 9.0).

HaCaT cells (34) were grown to confluency in 48-well plates in keratinocyte growth medium-2 (KGM-2) (PromoCell GmbH, Heidelberg, Germany) supplemented with medium supplement and calcium chloride solution provided by the supplier at 37°, 5% CO_2_. After confluence was reached, cells were incubated with different PI3Ki for 5 min. After incubation, 4 0μg/ml of EBA antibody solutions was added to the designated wells for 24 h. The following controls were included (antibody only, antibody/vehicle, normal human IgG only (Intratect 50 g/L, Biotest AG, Dreieich, Germany, the IgG was re-buffered before use to PBS using Amicon® Ultra-15 Centrifugal Filters), and normal human IgG/vehicle. Cells from two different passages were used and EBA-IgG from 2 donors each, leading to 4 data points.

The C5a content of the supernatants was measured using the Human Complement Component C5a DuoSet ELISA (R&D Systems, Minneapolis, USA) according to manufacturer instructions, with changes to detection using TMB One solution (Promega Corporation, Wisconsin, USA) and 0.9 M H_2_SO_4_ to stop the color reaction. IL-8 release was measured using the ELISA MAX™ human IL-8 standard set (Biolegend, San Diego, CA, USA) according to manufacturer instructions.

### PamGene Measurement

Kinase activity profiles were determined using the PamChip^®^ 4 protein tyrosine (PTK) peptide microarray system from PamGene International B.V. (BJ‘s-Hertogenbosch, The Netherlands). Human PMNs were diluted to a final concentration of 6.7 × 10^6^ cells/ml in RPMI 1640 (without phenol red) containing 5% FCS and pre-incubated with a final concentration of 5 μM AS-604850, 1 μM alpelisib, 10 μM TGX or vehicle (RPMI 1640 without phenol red/ 5%FCS, 0.1% DMSO) for 5 min (RT). These concentrations were selected as they led to a 50% reduction of ROS release in IC-stimulated PMNs. Flat bottom 6-well-plates were coated with immune complexes consisting of human COL7E-F antigen at a final concentration of 10 μg/ml and anti-human COL7 IgG1 antibody at a final concentration of 2 μg/ml (29), and 10^7^ PMNs/1.5 ml per well (containing the respective inhibitors or vehicle) were added. The plates were incubated at 37%, 5% CO_2_ for 0, 2, 8, or 15min, respectively. After incubation, the plates were placed on ice immediately, and the cell solution from each well was transferred to a separate 15 ml tube. Each well was also flushed with 1 ml of ice cold DPBS to detach cells adhered to the plate surface. The cell solutions were centrifuged for 5 min at 700 × g (4°C). The supernatant was discarded, and the cell pellet used to prepare lysates as described in the protocol provided by PamGene (Protocol #1160 for preparation of Lysates of Cell Lines or Purified Cells) (100 μl of lysis buffer per pellet). Protein concentrations in the lysates (above the recommended level >0.5 μg/μL) were determined using the Pierce BCA Protein Assay Kit (Waltham, MA, USA) according to the manufacturer‘s instructions (microplate procedure).

The Serine-Threonine Kinase (STK) and Protein Tyrosine Kinase (PTK) microarray assays were performed according to the manufacturer‘s instructions. Samples originating from the same donor and experimental setup (*n* = 3) were placed on the same microarray chip to enable more accurate comparisons. A FITC-conjugated anti-phosphotyrosine antibody was used for visualization during and after the pumping of lysates through the three-dimensional surface of the array. The capture of substrate phosphorylation signals was enabled by a computer-controlled CCD camera and measured repeatedly during a 1-h kinetic protocol using the Evolve software (PamGene International B.V.). The 1-h kinetic protocol showed a linear increase in the signal intensity for the majority of the peptide substrates, indicating that kinome profiling was run without methodological complications. The analysis of the images was performed using the BioNavigator Software (Ver. 6.3), with the predesigned protocols “STK Image Analysis,” “PTK Image Analysis,” “STK Basic Processing,” and “PTK Basic Processing.” Additional applications used were “Fit and apply a combat model”, “STK upstream kinase analysis,” and “PTK upstream kinase analysis.” After visual check and quality control, endpoint signal intensities minus background signals were calculated by BioNavigator for each spot representing each kinase peptide substrate per array. Subsequently, the data were log_2_-transformed before mean replicate signal intensity within each experiment was calculated for each peptide substrate. A kinase was considered to be modulated (either activated or inhibited) if it had a mean specificity score (= negative decadic logarithm of the likelihood of obtaining a higher difference between the groups when assigning peptides to kinases randomly) of 1 (*p* = 0.1) and a significance score (= likelihood of obtaining a higher difference for random assignment of values to treatment- and control groups) of 0.5 (*p* = 0.32). To detect changes caused by the tested compounds, samples for each timepoint and compound were compared with the control samples for the same timepoint. To ascertain the impact of the stimulation itself, control samples from before stimulation were compared to control samples at each timepoint (2, 8, and 15min). The mean kinase statistic (= calculated by averaging the difference between the signal intensity of a sample and its control value, normalized against a pooled estimate of the standard deviation in each sample, for each peptide assigned to a specific kinase) was used for further analysis:

Kinome Trees were created using Coral (35). Venn diagram analyses were performed in Venny2.0 (https://bioinfogp.cnb.csic.es/tools/venny/). Heatmaps were created using in R studio (Ver. 1.3.1093) the heatmap package (“pheatmap” Ver. 1.0.12). Enrichment analysis was performed using Webgestalt Gene Set Enrichment Analysis (36). Settings were as follows: minimum number of genes for a category: 3, maximum: 1000, Significance Level: Top 10. Number of Permutations: 1000. Pathway graphs were created using KEGG Mapper (Ver. 4.2) and sourced from KEGG (Release 95.2) or STRING database (37).

### Induction of Experimental EBA in Mice and Treatment Protocol

Rabbits were immunized with a fragment of murine type VII collagen NC1 (mCOL7^C^) in CFA/IFA by Eurogentec, Köln, Germany. Specific anti-mCOL7^C^ IgG from immune serum was isolated as previously described (26, 31, 38). Mice were injected in the ear base once with 30–100 μg of specific rabbit anti-mCOL7 IgG per mouse. The respective dose to induce a disease score of 25–50% of the affected ear surface area was determined in advance. PI3K inhibitors were administered orally (Supplementary Table 1), or topically applied to the ear 1 day prior to the initial anti-mCOL7^C^ IgG injection and was performed every day (in total 4 times). The ear thickness measurement, using a Mitutoyo 7301 dial thickness gauge (Neuss, Germany), and scoring was performed by a blinded person. For anesthesia, a mixture of 15 μg/g body weight Xylazin and 100 μg/g body weight Ketamin was injected i.p. into mice (26, 39).

### Histopathology and Direct Immunofluorescence Staining

Biopsies of lesional and perilesional skin were obtained on day 3 of the antibody transfer-induced EBA and prepared for examination by histopathology and immunofluorescence (IF) microscopy, as described previously (26, 31, 38). In brief, the biopsies collected from mice were fixed in 4% PBS-buffered formalin, and subsequently, sections of paraffin-embedded tissues were stained with hematoxylin and eosin. IgG deposits were detected by direct IF microscopy in frozen sections prepared from tissue biopsies using fluorescein isothiocyanate (FITC)-labeled antibodies specific to rabbit IgG (Dako, Glostrup, Denmark) as previously described (40).

### Statistical Analysis

Unless otherwise noted, data are presented as the mean ± SD or Tukey's box-and-whisker plots; the dots represent actual results for each sample. For comparisons of more than 2 groups, ANOVA was used. For equally distributed data, one-way ANOVA followed by a Bonferroni *t*-test for multiple comparisons was used; if the data were non-parametric, ANOVA on ranks (Kruskal-Wallis) was applied followed by a Bonferroni *t*-test for multiple comparisons. In all tests, *p* < 0.05 was considered indicative of significance. All statistical analyses were performed using SigmaPlot 13.0 (Systat Software, Erkrath, Germany).

For the *in vivo* studies a sample size calculation was performed. Assuming a minimal detectable difference in means of 0.5, a standard deviation of 0.25, and aiming at a power of 0.8 and an α of 0.05, 8 mice per group have to be included when using ANOVA with an appropriate post-test. Hence, 8 mice per group were included in the *in vivo* studies.

## Results

### IL-8-Induced Chemotaxis of PMNs Is Mainly Impaired by PI3Ki Selective for the β-, γ-, and δ-Isoforms

**Figure 1:**
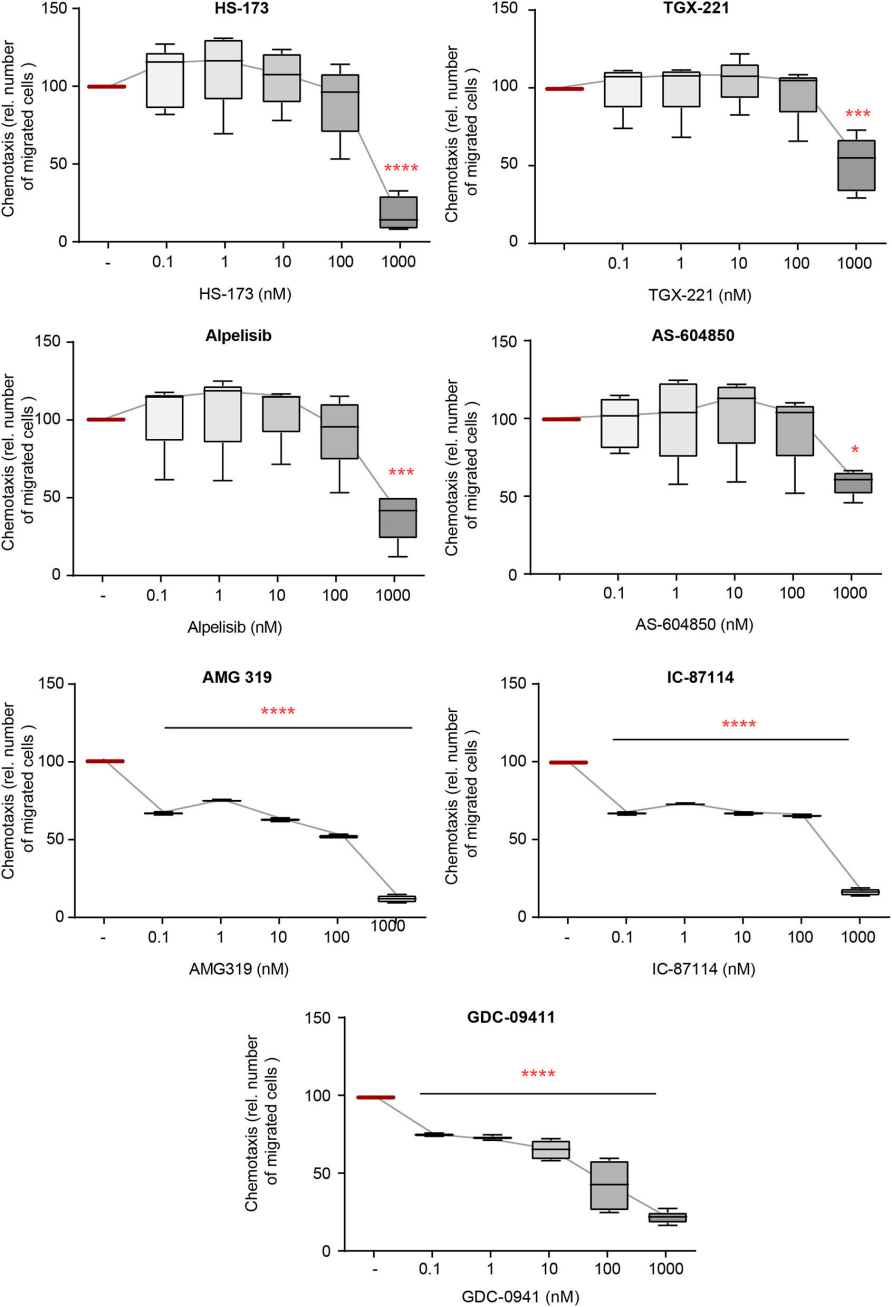
IL-8-induced chemotaxis from PMNs depends on PI3K. Chemotaxis of PMNs was induced by IL-8 in the presence of either one of the PI3K isoform-selective inhibitors. The attracted cell number during a time period of 60min is shown. Data were normalized to positive control (chemotaxis induced by IL-8 in the presence of solvent). Data are shown as Tukey's box-and-whisker plots. *n* = 5. ANOVA on ranks (Kruskal-Wallis) was applied followed by a Bonferroni *t*-test for multiple comparisons. ^*^p < 0.05, ^***^p < 0.001, ^****^p < 0.0001.

The directed chemotaxis of polymorphonuclear leukocytes (PMNs) to sites of local inflammation is an early-step in and a key prerequisite for their (patho)-physiological function in EBA (41). Within this multi-step process, cytokines, such as IL-8, are required to facilitate neutrophil extravasation, as well as their activation (42–44). We thus evaluated the impact of inhibition of the selected PI3Ki on IL8-induced PMN chemotaxis. All investigated inhibitors reduced IL-8-induced chemotaxis. PI3K inhibitors mainly selective for PI3Kδ- (AMG319 and IC87114) as well as PI3Kγ-selective (AS-604850 and AMG319) and PI3Kβ-selective inhibitors (TGX-221) inhibited chemotaxis at concentrations within the described IC_50_ values (Figure 1). Of note, the pan-PI3K inhibitor GDC-0941 had the most pronounced inhibitory effects on IL-8-induced neutrophil migration, with an efficacy at 0.1 nM. Also, PI3Kα-selective inhibitors (HS-173 and alpelisib) reduced chemotaxis at concentrations above the known IC_50_ values.

### PMN Adhesion and Spreading on ICs Are Predominantly Impaired by PI3Kδ- and PI3Kβ-Selective Inhibitors

**Figure 2:**
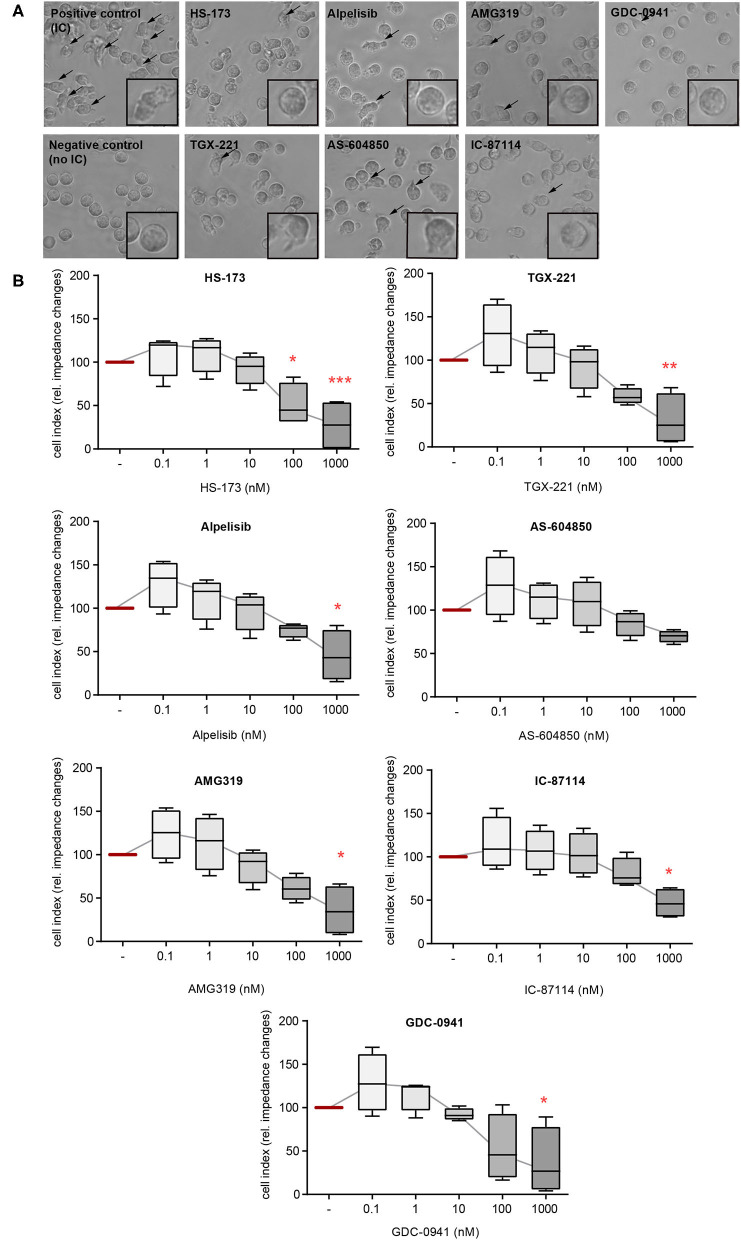
IC-induced spreading of PMNs mainly depends on PI3Kδ. Freshly isolated human blood PMNs were activated with immobilized ICs in the presence of either one of the PI3K isoform-selective inhibitors. **(A)** Microscopic appearance of PMNs 2 h after IC-stimulation. IC-stimulated PMNs show clear spreading on surface (arrows). The highest concentration of all inhibitors (1 μM) reduced the adhesion of PMNs. **(B)** Spreading was monitored live for 2 h and the area under curve (AUC) was analyzed. Data are shown as Tukey's box-and-whisker plots. *n* = 4. ANOVA on ranks (Kruskal-Wallis) was applied followed by a Bonferroni *t*-test for multiple comparisons. Data were normalized to IC-stimulated cells. ^*^p < 0.05, ^**^p < 0.01, ^***^p < 0.001.

In addition to ROS release, FcγR-dependent binding of neutrophils also induces their β2 integrin-dependent spreading and adhesion to IC-coated surfaces. This creates an enclosed space between the neutrophils and the target tissue, allowing a directional release of proteases and ROS. If neutrophil adhesion to IC-coated surfaces is blocked, the tissue is protected from the detrimental effects of proteases and ROS (30). We hence evaluated the impact of the PI3Ki on PMN spreading on IC-coated surfaces. Interestingly, PI3K inhibition had only marginal effects on IC-induced spreading and adhesion. Mostly, inhibitors with a PI3Kδ- and PI3Kβ-selectivity reduced PMN spreading (IC-87114, TGX-221, and AMG319, Figure 2). As AMG319 inhibits also p110γ, the effects on spreading could be also partially mediated by PI3Kγ. Yet, the second PI3Kγ-selective inhibitor (AS-604850) did not have an effect on IC-induced spreading of PMNs within the investigated concentration range. Furthermore, a statistically significant inhibition was observed for the p110α inhibitor HS-173 at concentrations of 0.1 and 1.0 μM. HS-173, GCD-0941 and alpelisib inhibited neutrophil spreading and adhesion only at the 1 μM dose, which are relative high doses (Figure 2).

### IC-Induced ROS Release From PMNs Is Sensitive to Blockade of All PI3K Isoforms

**Figure 3:**
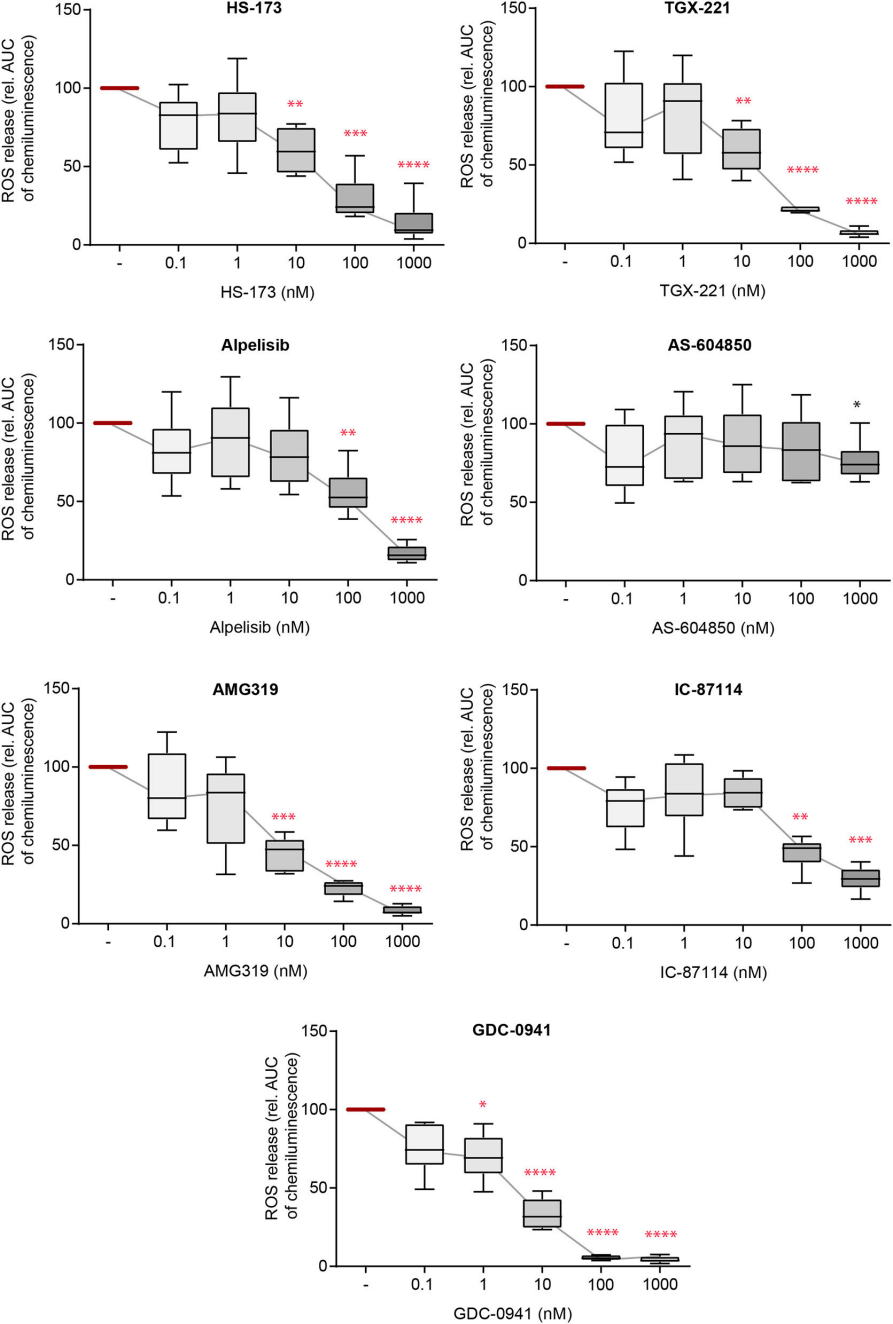
Immune complex (IC)-induced reactive oxygen species (ROS) release from PMNs depends on different PI3K isoforms. Human PMNs were activated with immobilized ICs in the presence of either one of the PI3K isoform-selective inhibitors. Release of ROS was tracked for 2 h and the area under curve (AUC) was calculated. Data were normalized to positive control (IC-stimulated PMNs with solvent). Data are shown as Tukey's box-and-whisker plots. *n* = 6. ANOVA on ranks (Kruskal-Wallis) was applied followed by a Bonferroni *t*-test for multiple comparisons. Data were normalized to stimulated cells. ^*^p < 0.05, ^**^p < 0.01, ^***^p < 0.001, ^****^p < 0.0001.

Activation of neutrophils and subsequent ROS release by FcγR-dependent binding to IC initiates local inflammation across many autoantibody-mediated diseases, including EBA (12, 45, 46). Herein, we thus used IC to activate PMN and evaluated the impact of selective PI3Ki on IC-induced ROS release. Here, all inhibitors, with the exception of PI3Kα subclass-selective inhibitors, impaired ROS release from IC-activated PMNs at doses close to the reported IC_50_ (Figure 3). The pan-PI3K inhibitor GDC-0941 had the most profound inhibitory effects, with efficacy at 1.0 nM. All effects were independent of any cytotoxic effects on neutrophils as none of the inhibitors effected Annexin V/propidium iodide staining in IC-stimulated neutrophils (Supplementary Figure 1).

### Selective Pharmacological Inhibition of PI3K Did Not Influence C5a and IL-8 Release From Keratinocytes

So far, we had demonstrated a selective contribution of PI3K to distinct neutrophil functions *in vitro*. To evaluate possible effects on other disease-relevant cell types, we stimulated human keratinocytes with EBA-IgG. We hence evaluated the impact of the PI3Ki on C5a and IL-8 release after 24 h of stimulation (Supplementary Figure 2) as complement activation and IL-8 release are key events in the effector phase of EBA and other pemphigoid diseases (21, 47–49). Compared to normal human IgG, EBA-IgG significantly increased IL-8 and C5a concentrations in the supernatants of HaCaT cells. Yet, none of the analyzed inhibitors had an inhibitory effect on EBA-IgG activated C5a release. Of note, after stimulation with EBA-IgG, several inhibitors (TGX-221, alpelisib, and IC-87115) showed a slight tendency even to increase the C5a release if used in low concentrations. Nevertheless, higher concentrations of the inhibitors did not affect C5a release. Interestingly, release of IL-8 was slightly inhibited by alpelisib at concentrations above the known IC_50_ values (1 μM) whereas none of the other inhibitors had any effect.

### Selective Pharmacological Inhibition of PI3K Reduced Inflammation in Experimental EBA

**Figure 4:**
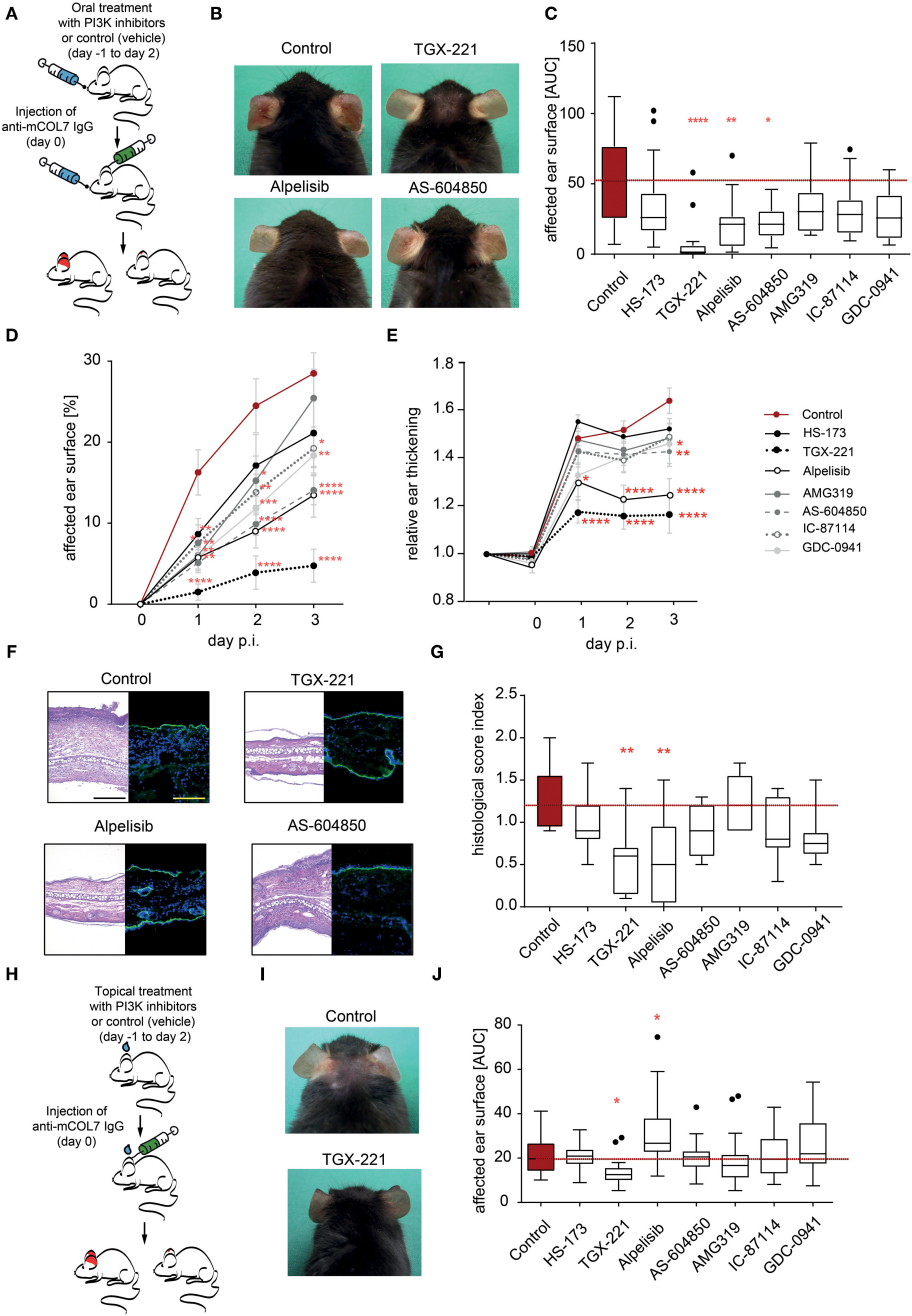
Treatment with TGX-221, alpelisib or AS-604850 impairs induction of experimental EBA. **(A)** Schematic illustration of experimental work flow. Anti-mCOL7 IgG was injected into the ears of C57BL/6J mice and the mice were orally treated with the indicated PI3K isoform selective inhibitors. **(B)** Representative clinical images of the mice on day 3. **(C)** The cumulative affected ear surface area (AUC) in mice treated with solvent or the indicated PI3K inhibitors. Data are shown as Tukey's box-and-whisker plots. ANOVA on ranks (Kruskal-Wallis) was applied followed by a Bonferroni t-test for multiple comparisons. **(D)** Percentage of ear surface area affected by EBA skin lesions at days 0–3 of the same experiment. Data are shown as mean ± SD. Two way-ANOVA with Bonferroni post test. **(E)** Relative ear swelling at days 0–3 of the same experiment. Data are normalized to day 0 of the experiment and shown as mean ± SD. Two way-ANOVA with Bonferroni post test. **(F)** Left panel: Representative H&E stained ear skin at day 3 after IgG-injection. Right panel: Representative direct IF microscopy of a skin biopsy, stained for anti-rabbit IgG (green) and nuclei (DAPI, blue). **(G)** The cumulative histological score index (AUC of skin infiltration, epidermal thickening and split formation at the DEJ) was analyzed in ear skin at day 3 of the experiment. Data are shown as Tukey's box-and-whisker plots. ANOVA on ranks (Kruskal–Wallis) was applied followed by a Bonferroni t-test for multiple comparisons. Data in panels **(C–E,G)** is based on 8 mice/group. **(H)** Schematic illustration of experimental work flow for topical treatment with PI3K isoform-selective inhibitors. Anti-mCOL7 IgG was injected into the ears of C57BL/6J mice and the mice were topically treated daily with the indicated PI3K isoform-selective inhibitors. **(I)** Representative clinical images on day 3 after topical treatment with solvent or TGX-221. **(J)** Cumulative affected ear surface area (AUC of percentage of day 0–3) in mice with topical PI3K inhibitor treatment. Data are shown as Tukey's box-and-whisker plots. ANOVA on ranks (Kruskal-Wallis) was applied followed by a Bonferroni t-test for multiple comparisons. Data in panels **(I,J)** is based on 8 mice/group. ^*^p < 0.05, ^**^p < 0.01, ^***^p < 0.001, ^****^p < 0.0001.

So far, we had demonstrated a selective contribution of PI3K to distinct neutrophil functions *in vitro*. Pathogenesis of inflammation in autoantibody-induced and neutrophil-dependent diseases, is, however, a multistep process that involves all (if not more) of the above tested steps (12). Furthermore, *in vivo* pharmacokinetics of the PI3Ki are also key determinants of their potential disease-modifying activity. Thus, prediction which PI3K inhibitor has anti-inflammatory effects in pre-clinical models of EBA is challenging. To address this, we used the mouse model of antibody transfer-induced EBA, in which disease manifestation depends on the presence of COL7 (auto)antibodies and a subsequent FcγR-dependent neutrophil activation (21). In a first set of experiments, mice, in which EBA was induced by anti-COL7-IgG injection, were orally treated with PI3K-selective inhibitors in a concentration that was recently shown to be effective known effective for other applications (50–56) (Figure 4). The p110α-selective inhibitor alpelisib and the p110γ-selective inhibitor AS-604850 showed impaired disease induction (ear thickness increase and affected ear surface area) by 50–60% (Figures 4D,E). Treatment with the p110β-selective inhibitor TGX-221 almost completely abolished the pathogenic activity of the anti-mCOL7 antibodies. A minor effect was observed for GDC-0941 and IC-87114 as visible by a reduced affected surface area (Figure 4D). All other tested inhibitors (HS-173 and AMG319) had no effect on clinical disease manifestation at the selected doses (Figures 4A–G).

### Topical Application of the PI3Kβ-Selective Inhibitor TGX-221 Impairs Induction of Experimental EBA

As systemic PI3K inhibition in humans may be associated with a relative high number of (serious) adverse events (27, 57), we next evaluated if topical application of the PI3Ki has an impact on disease manifestation in experimental EBA. Of the seven inhibitors, the p110β-selective inhibitor TGX-221 again profoundly impaired the induction of skin inflammation (Figures 4H–J). By contrast, all other PI3Ki did not reduce clinical disease manifestation in experimental EBA at selected concentration. During all experiments in the pre-clinical EBA mouse model, neither oral nor topical treatment with one of the PI3K inhibitors led to an increased suffering (weight reduction, general condition and behavior) in the mice (Supplementary Tables 2, 3).

### Kinome Analysis of Identified Unique Downstream Signaling Pathways of the Different, Disease-Modifying PI3K-Selective Inhibitors

**Figure 5:**
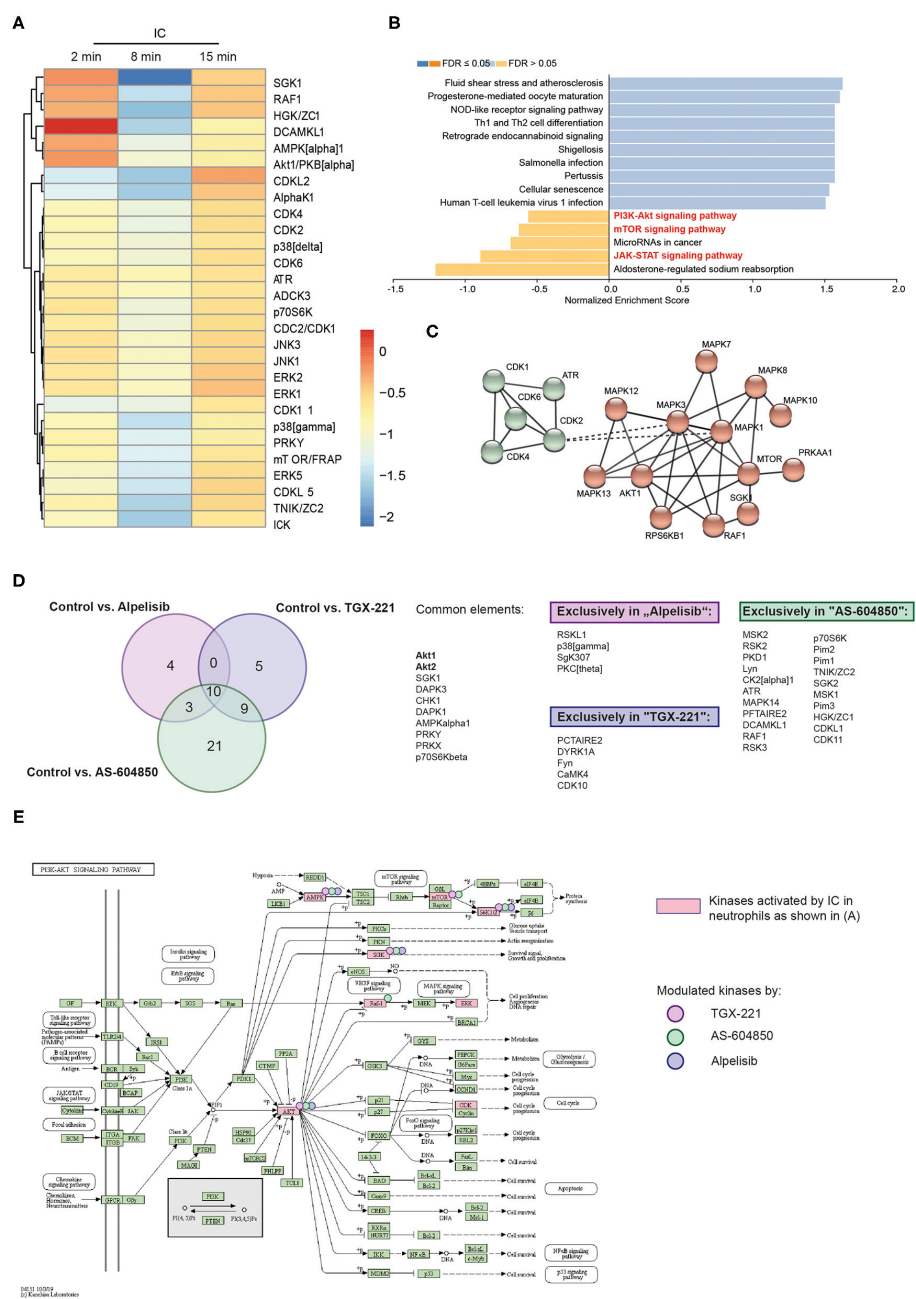
Unique impact of different PI3K inhibitors on downstream kinase activation. Freshly isolated human blood PMNs were activated with IC for 0, 2, 8, or 15min. Cells were lysed and the PTK and STK activity was measured by PamGene. **(A)** Heatmap of mean kinase statistic in comparison to unstimulated cells (0min). Blue: Kinase activity decreased/Red: Kinase activity increased. **(B)** Enriched KEGG pathways (as shown by Webgestalt) after IC-stimulation. **(C)** String network for IC-stimulated kinases (MCL clustering, inflation parameter 3) **(D)** Venn diagram and summary of the common and uniquely regulated kinase activities after use of three PI3K isoform-selective inhibitors that were effective in experimental EBA (alpelisib, AS-604850 and TGX-221). The comparison was performed for all time points with direct comparison to the kinase activity of ICs activated PMNs treated with solvent. **(E)** Influence of PI3K inhibition on PI3K/Akt and mTOR pathways, kinases regulated by ICs (cumulative stimulation for 2, 8, and 15min using PamGene) are shown in bright red. Kinases, that are regulated by the respective kinase inhibitors are marked with a reddish dot (TGX-221), green dot (AS-604850), and purple dot (alpelisib). Data is based on 3 replicates per group.

At this point, using the EBA mouse model, we had demonstrated that TGX-221, alpelisib or AS-604850 impaired induction of experimental EBA. To obtain more detailed insights into the orchestration of IC-induced signal transduction pathways selectively inhibited by these 3 therapeutically promising drugs, we performed a multiplex kinase activity profiling using PamGene (58). We incubated human PMNs with PI3Ki (alpelisib, AS-604850 or TGX-221) for 5 min and subsequently stimulated PMNs with IC for 0, 2, 8, or 15min. As expected, IC-stimulation of PMNs induced, among others, the PI3K/Akt, JAK/STAT and mTOR pathways (Figures 5A,B and Supplementary Table 4). In addition to these known pathways, we found a strong activity of several cyclin-dependent kinases (CDKs, Figure 5C). These are enriched in the KEGG pathway for “cellular senescence” (Supplementary Figure 3). Other relevant pathways that were regulated by IC-stimulation are summarized in Supplementary Figures 3, 4 (mTOR signaling, FoxO signaling and FcRγ-mediated phagocytosis).

To obtain more detailed insights into the mode of action of the effective inhibitors, we compared the kinase activity in PMNs after PI3K inhibition with the corresponding control time points (Figure 5D). Inhibition of PI3K by TGX-221 (Supplementary Figure 5), alpelisib (Supplementary Figure 6) or AS-604850 (Supplementary Figure 7) led to a decreased activity of Akt1/2 and the downstream kinases SGK, p70S6Kbeta (S6K1) and AMPK (Figures 5D,E). Interestingly, all 3 inhibitors also had unique effects on several kinases and KEGG pathways that were independent of the known PI3K network (Figure 5D). More specifically, inhibition using AS-604850 (Supplementary Figure 7) identified a total of 21 kinases that were exclusively de- or increased in their activity, indicating additional effects on different targets. By contrast, only 5 unique kinases were detected in TGX-221-treated PMN and 4 unique kinases in alpelisib treated PMNs (Figure 5 and Supplementary Tables 5–7).

## Discussion

We here systematically investigated the impact of seven PI3Ki with different isoform-selectivity on neutrophil functions *in vitro*, as well as their potential to reduce autoantibody-induced, neutrophil-driven inflammation in preclinical models of EBA. We demonstrate that the PI3Kβ-selective TGX-221 has the most pronounced disease-modifying activity, especially when applied topically. Of note, this superior *in vivo* effect of TGX-221 could not be derived from the *in vitro* characterization of the PI3Ki evaluated herein. More specifically, TGX-221 impaired IL-8-induced chemotaxis only at relatively high concentrations, while AMG 319 and IC-87114 dose-dependently and at quite low concentrations, reduced PMN chemotaxis. PMN spreading to IC, as well as IC-induced ROS release from PMN was equally well inhibited by HS-173, TGX-221, as well as several more PI3Ki in the IC-induced ROS release. Thus, *in vivo* disease models are required to demonstrate efficacy.

Hence, while this and previous (16, 18) reports demonstrate the fundamental role of PI3Kβ and PI3Kδ in the pathogenesis of skin inflammation in EBA, our data presented here does not rule out that other class I PI3K isoforms contribute to EBA pathogenesis—especially PI3Kα, one PI3Ki selective for this isoform (alpelisib) impaired induction of experimental EBA. Interestingly, alpelisib was blocking chemotaxis and ROS release only if used at rather high concentrations but in contrast reduced IL-8 release from human keratinocytes. Still, the kinase activation profiles indicate a specific inhibition of the PI3K pathway by alpelisib. In addition to these *in vitro* findings, the *in vivo* pharmacokinetics of a given drug are crucial for its activity. For alpelisib, over 50% are absorbed, with a T_(max)_ of 2 h and an elimination half-life from plasma of 13.7 h (59) which indicates a good bioavailability that could contribute to the effectivity of alpelisib in murine experimental EBA.

Dissection of the contribution of different PI3K isoforms to EBA pathogenesis, would require the use of PI3K isoform-deficient mice (1, 2, 60–62). We here, however, specifically focused on pharmacological PI3K inhibition allowing a better clinical translation.

**Figure 6:**
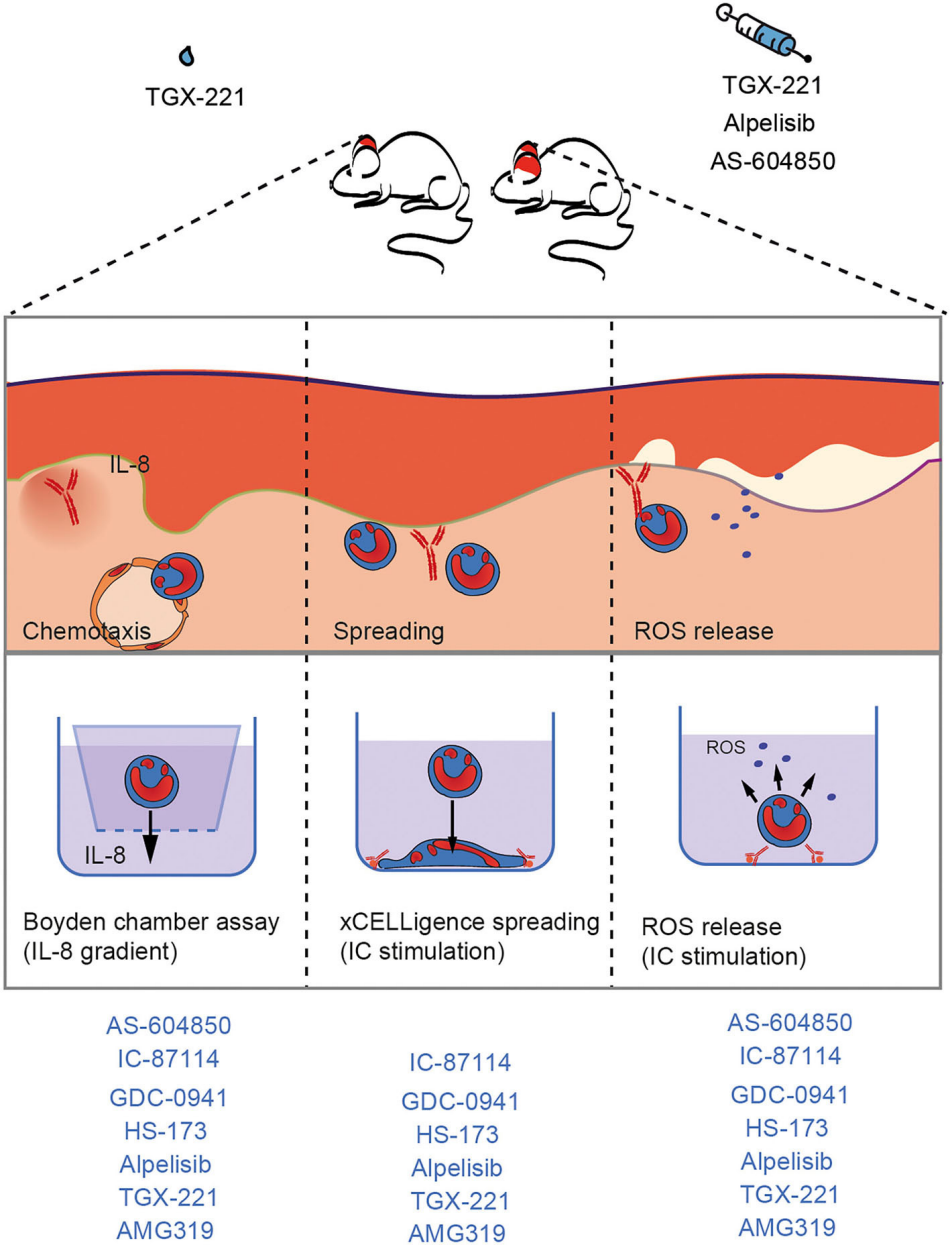
Unique impact of different PI3K inhibitors on neutrophil-dependent processes in EBA. In the pathogenesis of IC-induced EBA, PMN exert their pathogenic effects in a stepwise manner that is initiated by attracting them into the tissue (chemotaxis), spreading, and adhesion to surfaces of IC deposits and ultimately results in the release of pro-inflammatory substances, such as ROS. As these processes strongly depend to PI3K, inhibition leads to improvement of EBA.

In the pathogenesis of the inflammatory type of EBA, PMNs exert their pathogenic effects in a stepwise manner that is initiated by attracting them into the tissue (chemotaxis) and ultimately results in the release of pro-inflammatory substances, such as ROS (Figure 6). Extravasation of PMNs to sites of inflammation is an early key step driving EBA (63, 64). Within this multi-step process, cytokines, such as IL-8, are required to facilitate neutrophil chemotaxis, as well as activation (42–44). We here demonstrate that PI3Kγ- and PI3Kδ- selective inhibitors block PMN chemotaxis. By contrast, selective inhibitors of PI3Kα have no major impact on IL-8-induced PMN migration. These findings support earlier notions demonstrating that in fMLP-stimulated neutrophils, the PI3Kδ inhibitor IC-87114 affected polarized morphology of neutrophils, PIP_3_ production and chemotaxis, but did not block F-actin synthesis or neutrophil adhesion (65). Regarding PI3Kγ, G-protein-coupled receptor (GPCR) stimulation of PI3Kγ induced PMN migration (and the oxidative burst) (66). Therefore, both PI3Kγ and δ seem to be important to mediate PMN chemotaxis across several stimuli.

By creating a protected space between the neutrophil and the target tissue, neutrophil spreading and adhesion to surfaces of IC deposits is a prerequisite for autoantibody-induced tissue damage (30). This process is regulated by PI3K activation (67, 68). Previous data demonstrated that the pan-PI3K inhibitor wortmannin or the PI3Kα/β/δ inhibitor LY294002 impaired neutrophil binding to ICAM-1 after fMLP stimulation (69). Other data on the PI3K isoforms involved in these processes is sparse. PI3Kγ has been shown to mediate the transition from rolling to firm adhesion, a process depending on β2 integrins (70). We here show that β2 integrin-dependent neutrophil adhesion to immobilized IC mostly depends on PI3Kγ /δ.

Regarding ROS release from IC-activated neutrophils, we here show that this requires PI3Kβ, γ, and δ. These findings are consisted with published data for PI3Kβ and δ (16, 18), which may even act synergistically in this context (16). Interestingly, while important for TNF/fMLP-induced ROS release from neutrophils (19), as shown here, PI3Kγ-selective inhibition only has a marginal impact on IC-induced ROS release. In line with this, pan-PI3K inhibition completely abolished the IC-induced ROS release.

All of the above neutrophil functions contribute to local inflammation in EBA (21). Yet, the *in vitro* inhibitory effects of PI3K isoform-selective drugs do not allow to predict their impact on the complex disease pathogenesis *in vivo*. To investigate if inhibition of specific PI3K isoforms has an impact on *in vivo* inflammation provoked by tissue-bound IC, we used the antibody transfer model of EBA. We specifically selected a skin inflammation mouse model because topical application, which presumably leads to less adverse events, is possible in chronic inflammatory skin diseases. Out of 7 tested PI3K inhibitors, AS-604850 (PI3Kγ-selective), alpelisib (PI3Kα-selective) and TGX-221 (PI3Kß-selective) showed a significant inhibition of ear thickening and led to a more than 50% reduction of the affected ear surface area. One of these inhibitors, TGX-221 was even effective if applied topically. Given that EBA is notoriously difficult to treat, achieving remissions after 9 month of systemic immunosuppressive treatment (23), addition of a (preferably topically applied) PI3Ki could potentially lead to a more rapid induction of remission.

Previously, we had documented that induction of skin inflammation is hampered in PI3Kβ-deficient mice or in mice treated with a PI3Kδ-selective inhibitor (16, 18). Overall, these data and the results presented herein point toward a non-redundant role of these 2 class I PI3K isoforms.

Next, we here evaluated the impact of the three PI3K isoform-specific inhibitors on downstream signaling cascades, allowing detailed insights into PI3K isoform-specific signaling pathways in human PMNs, which may provide additional and more downstream targets to block IC-induced PMN activation. Unbiased analysis of kinase activity in IC-activated PMN treated with either TGX-221, alpelisib or AS-604850 identified a number of shared downstream kinases like Akt1/2 and the downstream kinases SGK, p70S6Kbeta (S6K1) and AMPK. However, the majority of changes in kinase activity after treatment of PMN with either of the compounds was specific for each inhibitor, and most pronounced for AS-604850.

Collectively, we here defined the differential impact of PI3K isoforms on immune complex-induced neutrophil signaling and function, and identify the PI3Kβ-selective TGX-221 as a potential topical treatment for the inflammatory type of EBA and other autoimmune skin blistering diseases with a similar pathogenesis.

## Data Availability Statement

The original contributions presented in the study are included in the article/Supplementary Material, further inquiries can be directed to the corresponding author/s.

## Ethics Statement

The studies involving human participants were reviewed and approved by University of Lübeck, Lübeck, Germany, AZ 09-140. The patients/participants provided their written informed consent to participate in this study. The animal study was reviewed and approved by Animal Care and Use Committee (Kiel, Germany)—AZ122.5(108/08-15).

## Author Contributions

RL and KBi: conceptualization and methodology and writing—original draft. HZ, AK, NG, CO, CH, MR, VH, NE, and RV: investigation. KBi and CO: data curation. RL: funding acquisition and project administration. RL, GV, FP, TL, and XY: resources. MB-H, XY, FP, RL, and KBi: supervision. HZ, AK, CO, NG, MR, CH, VH, MB-H, NE, KBo, GV, RV, TL, XY, FP, RL, and KBi: writing—review and editing. All authors contributed to the article and approved the submitted version.

## Funding

This study was funded by Research Training Group Modulation of Autoimmunity (GRK 1727), Clinical Research Unit Pemphigoid Diseases – Molecular Pathways and their Therapeutic Potential (KFO303), and Cluster of Excellence Precision Medicine in Chronic Inflammation (EXC2167), all from the Deutsche Forschungsgemeinschaft (DFG). RL was supported by the Schleswig-Holstein Excellence-Chair Program (State of Schleswig-Holstein).

## Conflict of Interest

RL has received honoraria and research grants from the following companies: Admirx, Almirall, Amryth, ArgenX, Biotest, Biogen, Euroimmun, Incyte, Immungenetics, Lilly, Novartis, UCB Pharma, Topadur, True North Therapeutics, and Tx Cell. The remaining authors declare that the research was conducted in the absence of any commercial or financial relationships that could be construed as a potential conflict of interest.

## Publisher's Note

All claims expressed in this article are solely those of the authors and do not necessarily represent those of their affiliated organizations, or those of the publisher, the editors and the reviewers. Any product that may be evaluated in this article, or claim that may be made by its manufacturer, is not guaranteed or endorsed by the publisher.
