# Supplementary material for: Topical Application of the PI3Kβ-Selective Small Molecule Inhibitor TGX-221 Is an Effective Treatment Option for Experimental Epidermolysis Bullosa Acquisita
Source: Front Med (Lausanne). 2021 Sep 7;8:713312. doi: 10.3389/fmed.2021.713312 (PMC8452940; doi:10.3389/fmed.2021.713312)
Supplement: Supplementary file 1 [file Data_Sheet_1.pdf]

Supplementary Figures

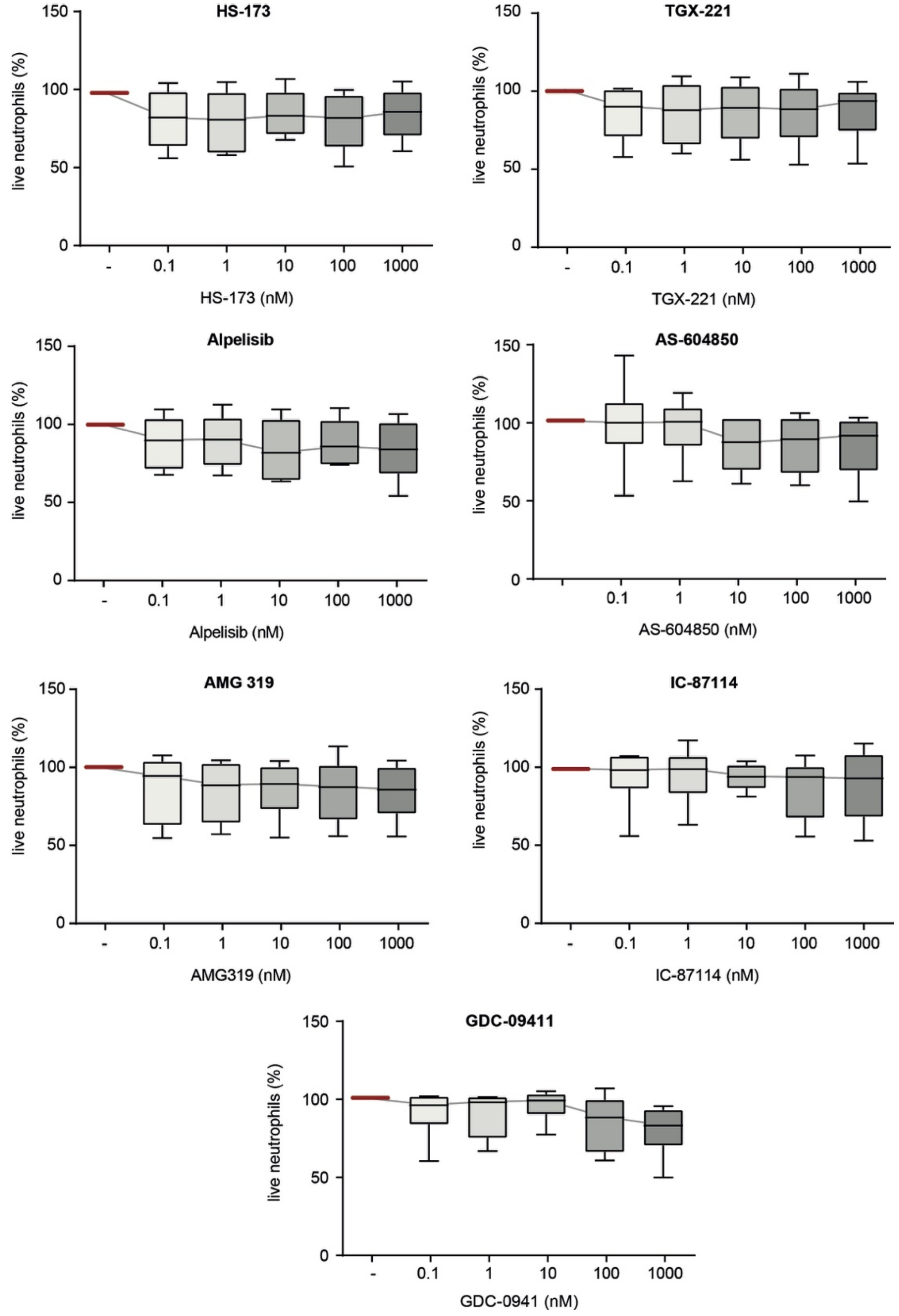

Figure S1. None of the PI3K inhibitors had toxic effects on human PMNs. Human PMNs were stimulated with immobilized ICs in presence/absence of either one of the PI3K isoform-selective inhibitors. To exclude toxicity by the inhibitors, the amount of propidium iodide (PI)- and Annexin V-positive cells after IC stimulation was identified. Data were normalized to positive control (IC-stimulated PMNs with solvent). Data are shown as Tukey's box-and-whisker plots. n=6. ANOVA on ranks (Kruskal-Wallis) was applied followed by a Bonferroni t-test for multiple comparisons. Data were normalized to stimulated cells. *$p<0.05$, **$p<0.01$, ***$p<0.001$, ****$p<0.0001$.

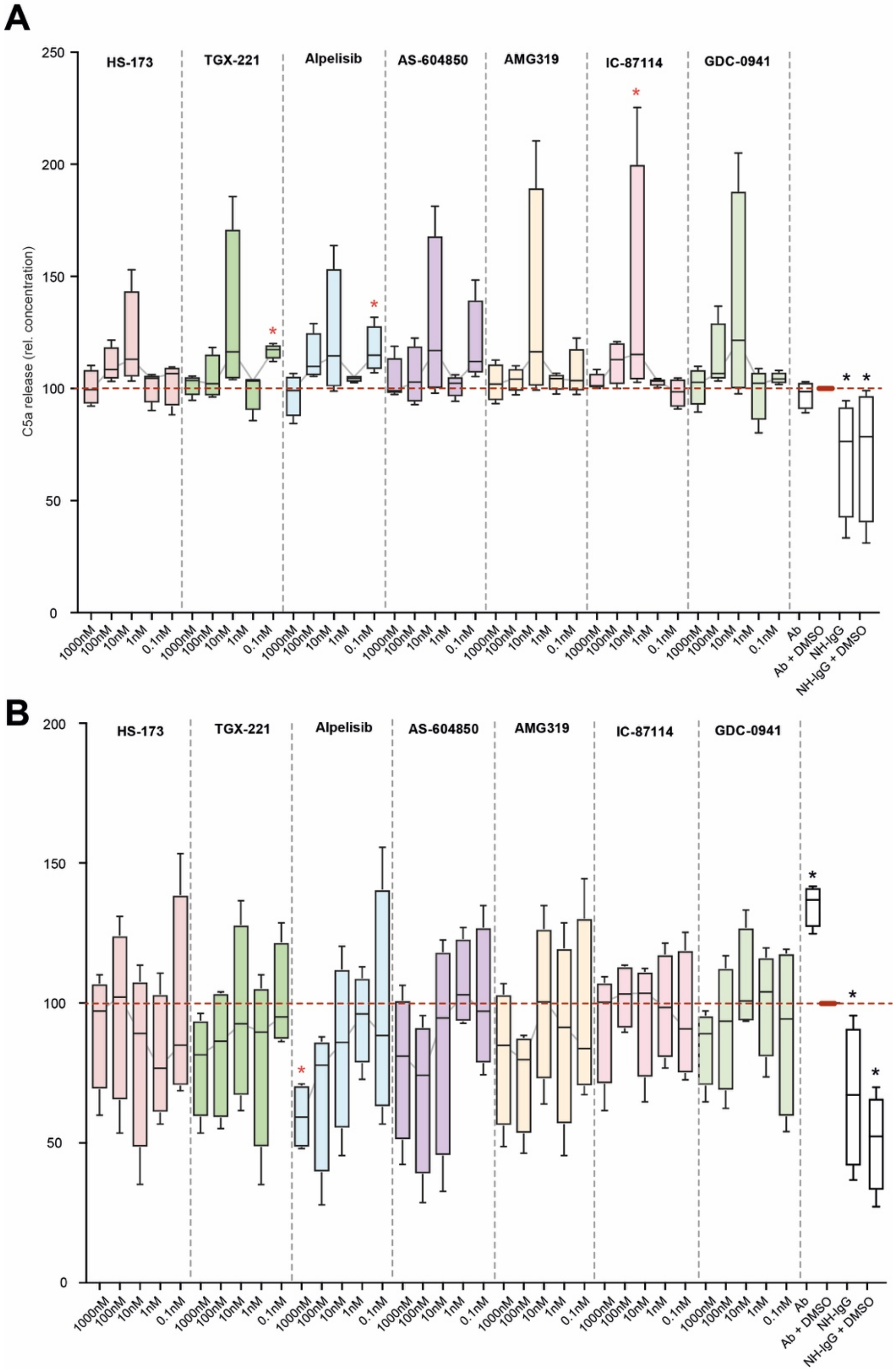

Figure S2. EBA-IgG-induced C5a and IL-8 release from keratinocytes is mostly independent of PI3K. HaCaT cells were stimulated for 24 h with purified EBA-IgG in presence/absence of either one of the PI3K isoform-selective inhibitors and the **(A)** C5a and **(B)** IL-8 release into the supernatant was determined. Data were normalized to positive control (stimulation of cells with EBA-IgG and solvent). Data are shown as Tukey's box-and-whisker plots. n=4. Red asterisks: ANOVA on ranks (Kruskal-Wallis) was applied followed by a Bonferroni t-test for multiple comparisons individually for every PI3Ki in comparison to stimulated cells (Ab + DMSO). Incubation of cells with normal human IgG (NH-IgG) (± vehicle) served as negative control. Black asterisks: Mann-Whitney test was applied for comparison between positive control (Ab + DMSO) and different negative controls. Data were normalized to stimulated cells. *$p<0.05$, **$p<0.01$, ***$p<0.001$, ****$p<0.0001$.

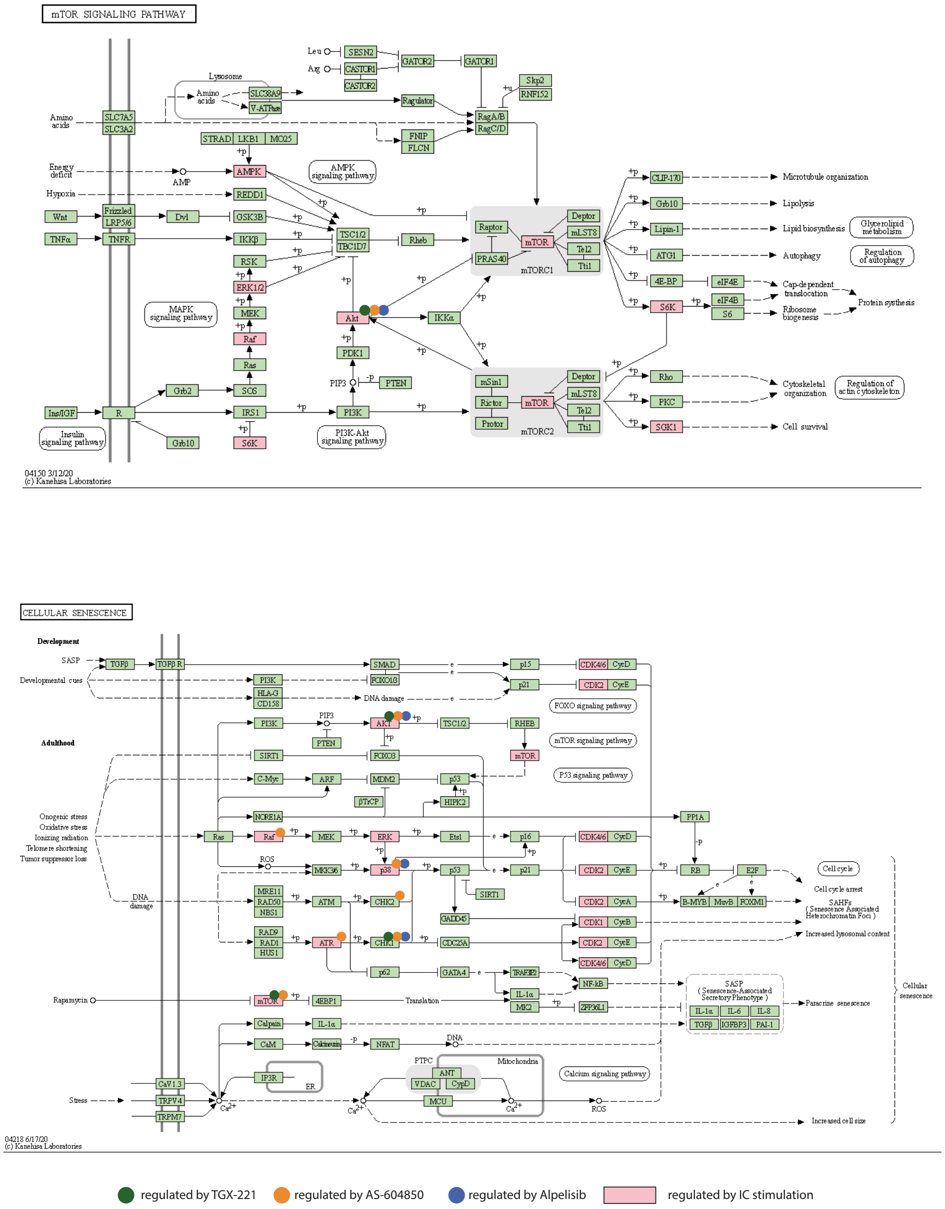

Figure S3. Influence of PI3K inhibition on mTOR pathways and pathways for cellular senescence. KEGG pathways for cellular senescence (https://www.genome.jp/dbget-bin/www_bget?hsa04218) and mTOR signaling pathway (https://www.genome.jp/kegg-bin/show_pathway?hsa04150), kinases regulated by ICs (cumulative stimulation for 2, 8, and 15 min using PamGene) are shown in bright red. Kinases, that are regulated by the respective kinase inhibitors are marked with a green dot (TGX-221), orange dot (AS-604850) and blue dot (alpelisib).

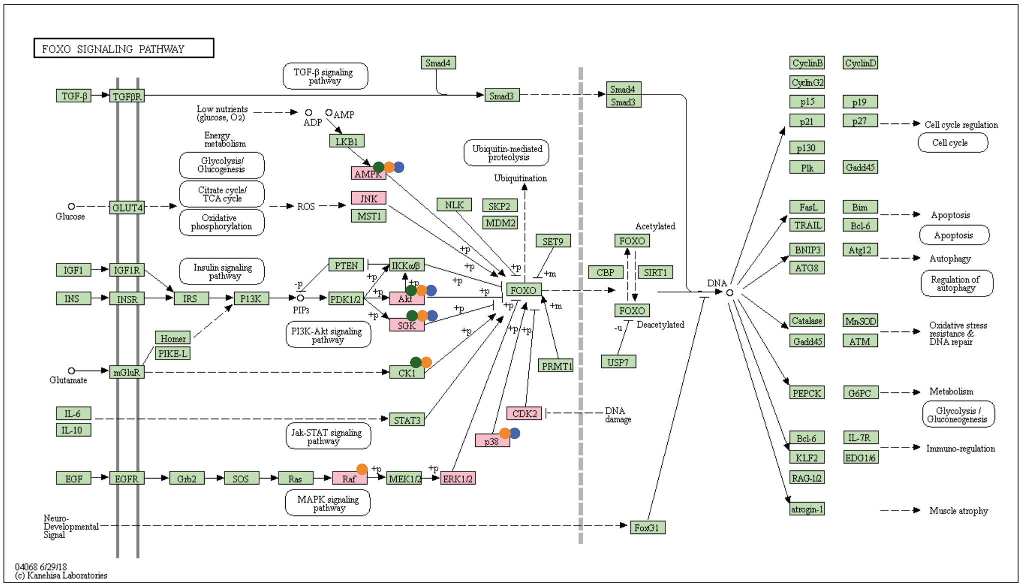

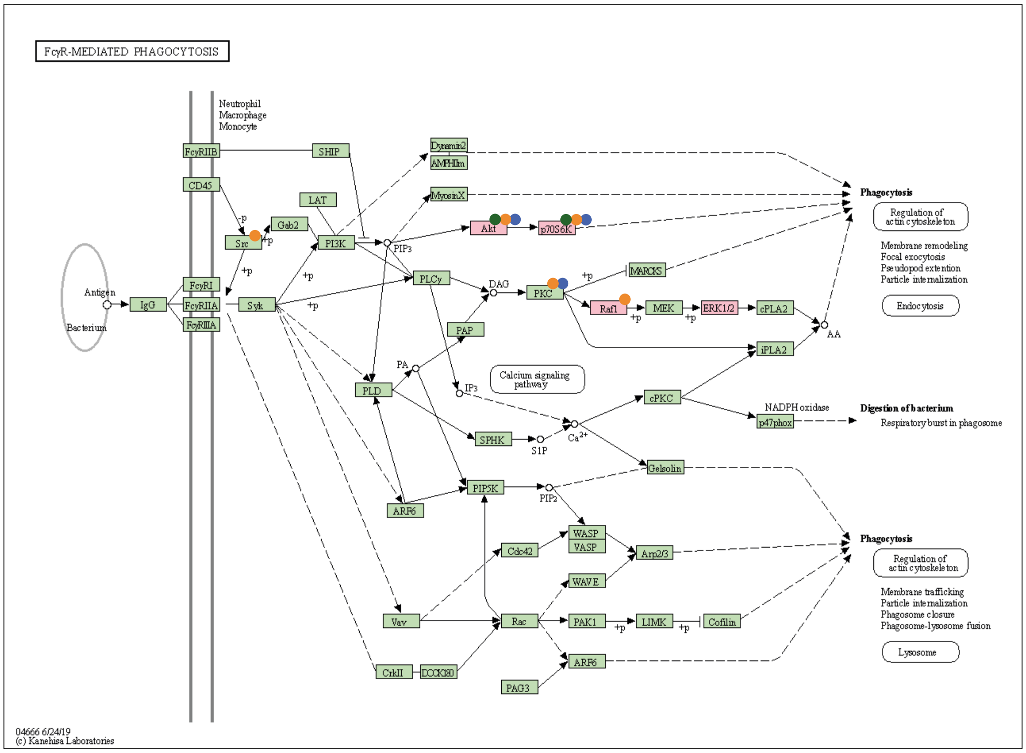

regulated by TGX-221 regulated by AS-604850 regulated by Alpelisib regulated by IC stimulation

Figure S4. Influence of PI3K inhibition on KEGG pathways for FOXO signaling and FcγR-mediated phagocytosis. KEGG pathways for FOXO signaling (https://www.genome.jp/kegg-bin/show_pathway?hsa04068) and FcγR-mediated phagocytosis signaling pathway (https://www.genome.jp/kegg-bin/show_pathway?hsa04666), kinases regulated by ICs (cumulative stimulation for 2, 8, and 15 min using PamGene) are shown in bright red. kinases regulated by ICs (cumulative stimulation for 2, 8, and 15 min using PamGene) are shown in bright red. Kinases, that are regulated by the respective kinase inhibitors are marked with a green dot (TGX-221), orange dot (AS-604850) and blue dot (alpelisib).

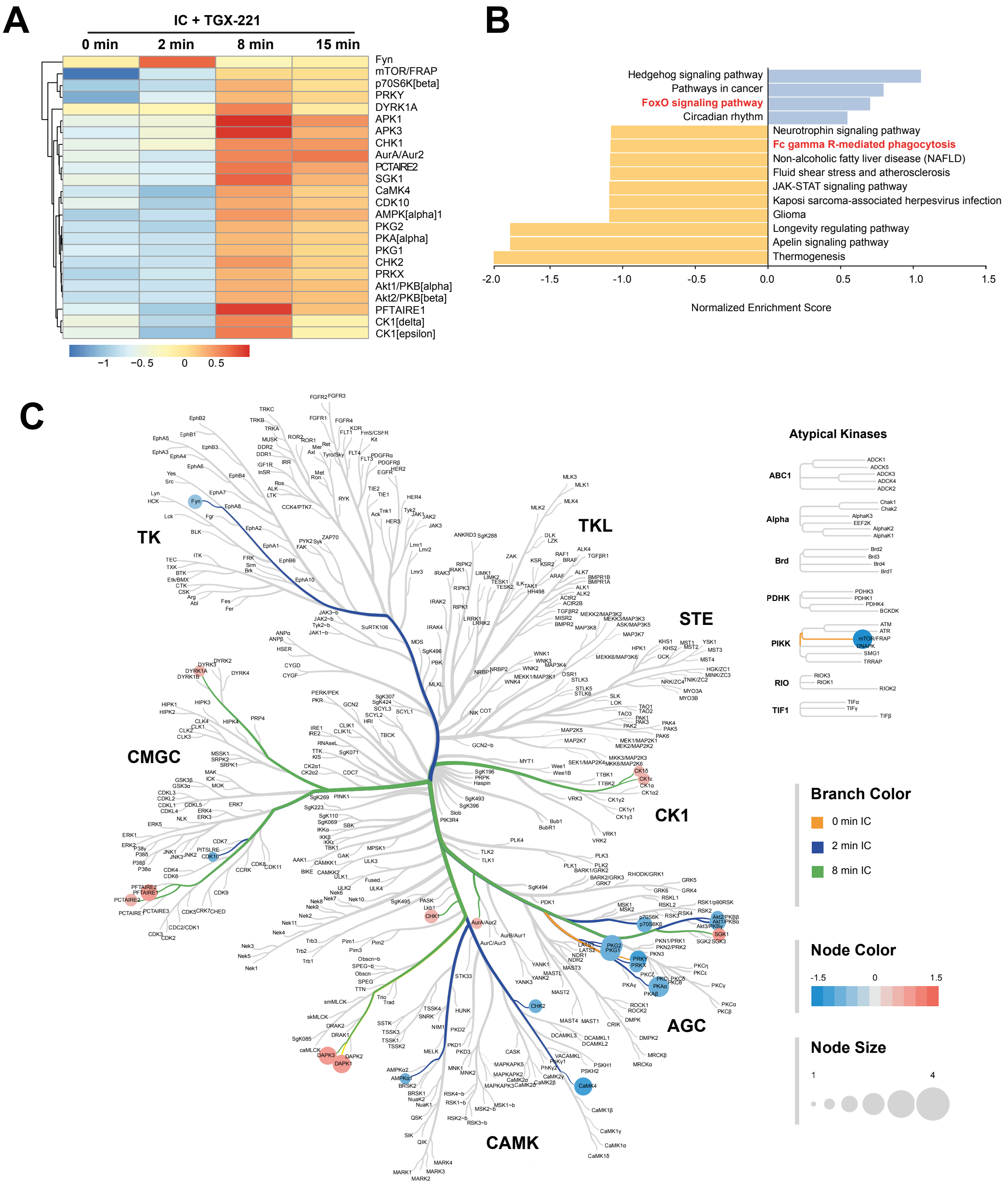

Figure S5. Inhibition of IC-induced signal transduction by TGX-221. Freshly isolated human blood PMNs were treated with w/ or w/o 10 µM TGX-221 for 5 min and subsequently activated with IC for 0, 2, 8, or 15 min. Cells were lysed and the PTK and STK activity was measured by PamGene. (A) Heatmap of mean kinase statistic in comparison to the respective timepoints in IC-stimulted PMNs. (B) Enriched KEGG pathways (as shown by Webgestalt). (C) Kinome tree of all regulated PKT and STKs over the time. Node size: mean final score, node color: mean kinase statistic. Data is based on 3 replicates per group.

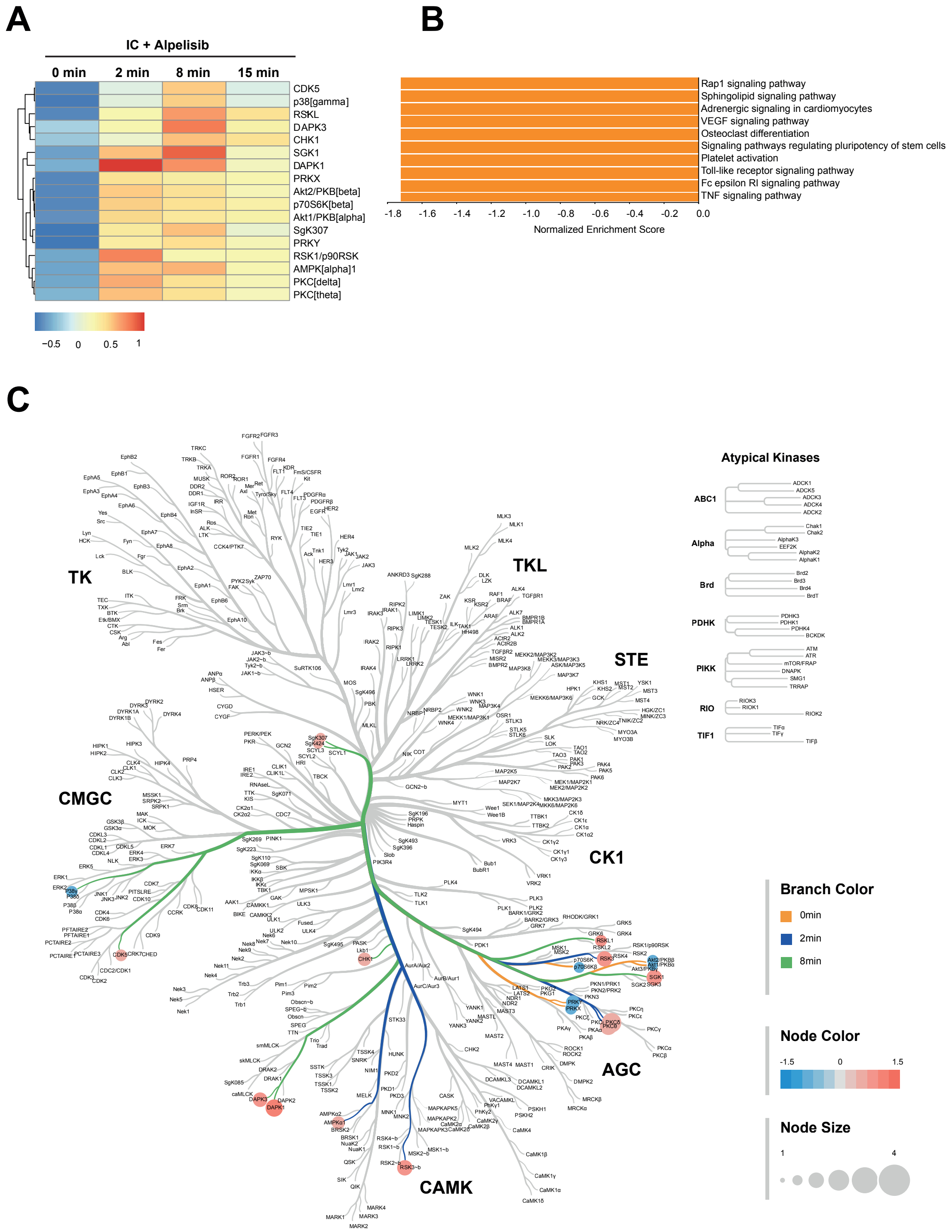

Figure S6. Inhibition of IC-induced signal transduction by alpelisib. Freshly isolated human blood PMNs were treated with w/o 1 µM alpelisib for 5 min and subsequently activated with IC for 0, 2, 8, or 15 min. Cells were lysed and the PTK and STK activity was measured by PamGene. (A) Heatmap of mean kinase statistic in comparison to the respective timepoints in IC-stimulted PMNs. (B) Enriched KEGG pathways (as shown by Webgestalt). (C) Kinome tree of all regulated PKT and STKs over the time. Node size: mean final score, node color: mean kinase statistic. Data is based on 3 replicates per group.

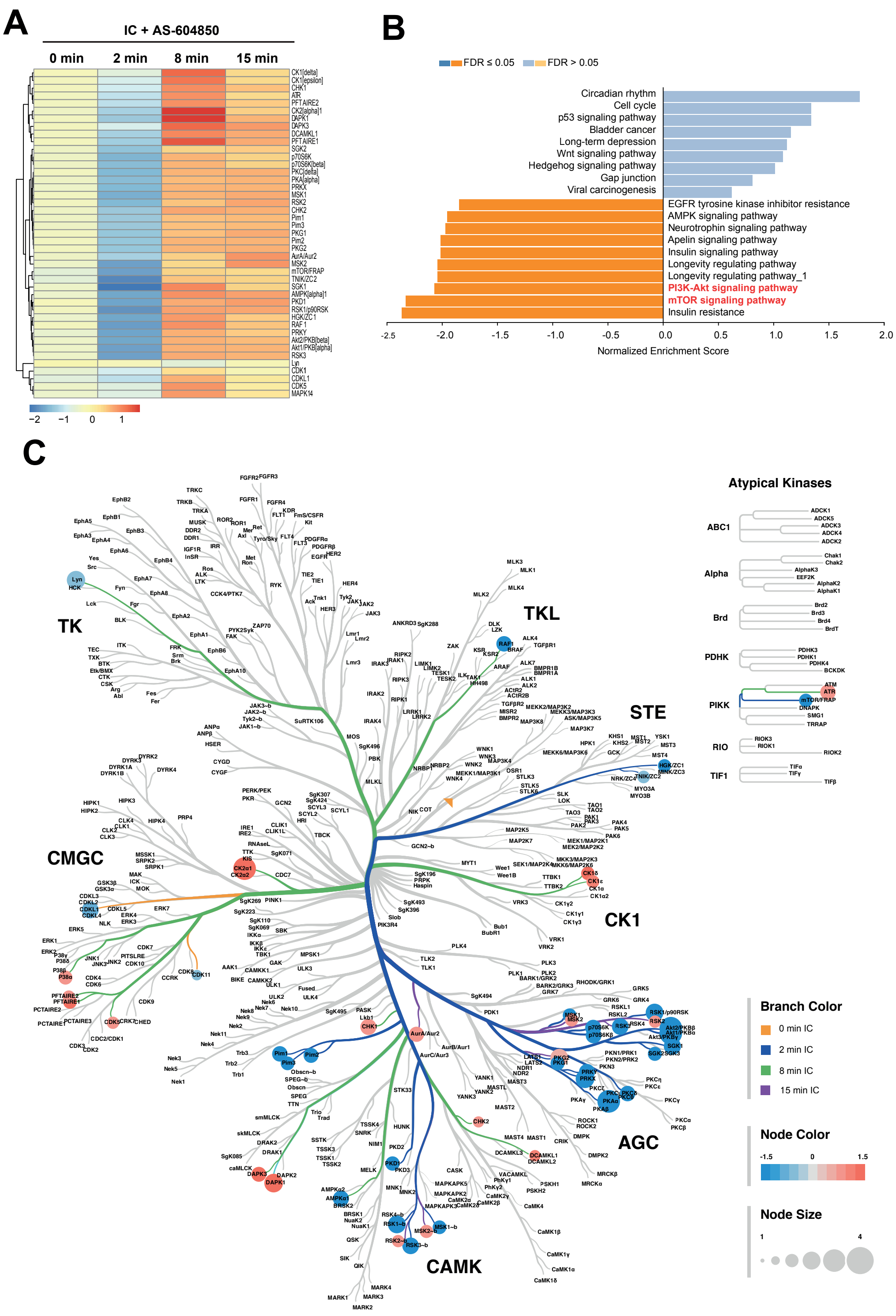

Figure S7. Inhibition of IC-induced signal transduction by AS-604850. Freshly isolated human blood PMNs were activated with IC for 0, 2, 8, or 15 min w/o 5 μM alpelisib. Cells were lysed and the PTK and STK activity was measured by PameGene. (A) Heatmap of mean kinase statistic in comparison to the respective timepoints in IC-stimulted PMNs. (B) Enriched KEGG pathways (as shown by Webgestalt). (C) Kinome tree of all regulated PKT and STKs over the time. Node size: mean final score, node color: mean kinase statistic. Data is based on 3 replicates per group.

Supplementary Tables

Name	IC50 p110α	IC50 p110β	IC50 p110γ	IC50 p110δ	Catalog no.	Stock (DMSO)	Dosage /day	MW	Reference Citation
HS-173	0.8 nM	-	-	-	S7356	25 mg/mL	20 mg/kg	422.46	(Camps et al., 2005)
alpelisib	5.0 nM	-	-	-	S2814	62.5 mg/mL	50 mg/kg	441.47	(Junttila et al., 2009)
TGX-221	5.0 µM	5.0 nM	>10 µM	0.1 µM	S1169	312.5 mg/mL	250 mg/kg	364.4	(Soond et al., 2010)
AS-604850	4.5 µM	>20 µM	0.55 µM	>20 µM	S2681	37.5 mg/mL	30 mg/kg	285.2	(Bird et al., 2011)
AMG319	33 µM	2.7 µM	850 nM	18 nM	S7813	3.75 mg/mL	3 mg/kg	385.4	(Son et al., 2013)
IC-87114	-	75 µM	29 µM	0.5 µM	S1268	75 mg/mL	60 mg/kg	397.43	(Cushing et al., 2015)
GDC-0941	3.0 nM	33 nM	75 nM	3.0 nM	S1065	187.5 mg/mL	150 mg/kg	513.64	(Yuan et al., 2019)

Table S1. Overview of the used PI3K isoform-selective inhibitors. Numbers correspond to the IC50 in nM, and if in bold, indicate the selectivity of the compound. "-" indicates that no data on the IC50 for that isoform is provided. All IC_{50}s were determined in cell-free assays. IC50 values in the detected range (<1 nM - 1 µM) for performed in vitro experiments are indicated in bright green. Data has been derived from the Selleckchem Website (https://www.selleckchem.com/, accessed April 16th, 2021).

I Body weight	value
unaffected or rise	0
Weight reduction <5 % compared to day 0 of the experiment	1
Weight reduction 5-10 % compared to day 0 of the experiment	5
Weight reduction 11-20 % compared to day 0 of the experiment	10
Weight reduction >20 % compared to day 0 of the experiment	20
II General condition	
Coat smooth, shiny, body openings clean, eyes clear, shiny	0
Fur blunt, disorderly, unkempt body openings, eyes cloudy, increased muscle tone	1
Dirty coat, sticky or wet body openings, abnormal posture, eyes cloudy, increased muscle tone	5
Dirty coat, sticky or damp body openings, abnormal posture, eyes cloudy; high muscle tone	10
Cramps, paralysis (trunk muscles, extremities),wheezing, animal feels cold	20
III Spontaneous behavior	
Normal behavior (sleeping, reaction to blowing and touching, curiosity, social contacts)	0
Small deviations from the normal behavior	1
Unusual behavior, impaired motor function or hyperkinetics (noticeable scratching over 2 of 10 min)	5
Self-isolation, lethargy, pronounced hyperkinetics or behavioral stereotypies, coordination disorders hyperkinetics (noticeable scratching behavior over 5 of 10 min)	10
Repeated pain sounds when grasping, self-amputation (autoaggression, autotomy)	20
IV Clinical findings (measurements of temperature and respiration only if abnormalities are observed under II and III)	
Temperature, respiration and pulse normal, extremities warm, mucous membranes well supplied with blood	0
Small deviations from the normal situation	1
Temperature deviation 1 - 2 °C, respiration and pulse + 30%	5
Temperature deviation> 2 °C, respiration / pulse +/- 50%	10
Temperature, respiration and pulse normal, extremities warm, mucous membranes well supplied with blood	20
Sum	0-80

Table S2. Adverse events score (I). The sum of all side adverse effects was added to a total sum showed in supplemental table 3.

Systemic treatment	Adverse events score (mean ± SD)
HS-173	2.50 ± 2.20
alpelisib	3.13 ± 3.60
TGX-221	1.50 ± 1.41
AS-604850	1.25 ± 0.46
AMG319	1.25 ± 0.46
IC-87114	3.50 ± 4.63
GDC-0941	1.13 ± 0.35
control	1.56 ± 1.74
**Topical treatment**	**Adverse events score (mean ± SD)**
HS-173	2.75 ± 2.05
alpelisib	6.00 ± 3.78
TGX-221	2.63 ± 2.13
AS-604850	3.88 ± 3.52
AMG319	1.25 ± 0.71
IC-87114	3.00 ± 2.51
GDC-0941	2.38 ± 2.26
control	3.44 ± 3.50

Table S3. Adverse events score (II). The mean and SD of the adverse effects of the experiments shown in figure 5 are summarized in here. Treatment with PI3K inhibitors did not increase the suffering of mice during local EBA as the adverse events score is not significantly changed.

Kinase	2 min	8 min	15 min
TNIK/ZC2	-0.771	-1.545	-0.514
SGK1	-0.174	-2.114	-0.465
RAF1	-0.264	-1.460	-0.446
PRKY	-0.796	-1.297	-0.754
p70S6K	-0.608	-1.103	-0.561
p38[gamma]	-0.877	-1.454	-0.745
p38[delta]	-0.857	-1.086	-0.641
mTOR/FRAP	-0.773	-1.300	-0.630
JNK3	-0.572	-0.956	-0.511
JNK1	-0.573	-0.964	-0.519
ICK	-0.924	-1.610	-0.597
HGK/ZC1	-0.319	-1.702	-0.427
ERK5	-0.965	-1.355	-0.547
ERK2	-0.619	-0.990	-0.431
ERK1	-0.626	-0.951	-0.406
DCAMKL1	0.259	-1.562	-0.767
CDKL5	-0.992	-1.395	-0.482
CDKL2	-1.372	-1.625	-0.250
CDK6	-0.900	-1.087	-0.560
CDK4	-0.861	-1.157	-0.544
CDK2	-0.813	-1.181	-0.535
CDK11	-1.202	-1.183	-0.619
CDC2/CDK1	-0.674	-1.095	-0.541
ATR	-0.680	-0.848	-0.582
AMPK[alpha]1	-0.269	-1.168	-0.757
AlphaK1	-1.279	-1.612	-0.427
Akt1/PKB[alpha]	-0.204	-1.068	-0.739
ADCK3	-0.671	-0.925	-0.631

Table S4. Mean kinase statistic of PKT and STK activation. Human PMNs (n=3) were stimulated with hCOL7/anti-hCOL7 ICs for the indicated time points. The mean kinase statistic is shown here as grey if upregulated (positive value) or downregulated (negative value) in comparison to unstimulated PMNs if they had a mean specificity score of 1 and a significance score of 0.5.

Kinase	0 min	2 min	8 min	15 min
SGK1	-0.506	-0.654	0.695	0.319
PRKY	-1.101	-0.632	0.306	0.185
PRKX	-0.847	-0.701	0.312	0.187
PKG2	-0.746	-0.870	0.317	0.195
PKG1	-0.673	-0.777	0.284	0.162
PKA[alpha]	-0.737	-0.746	0.305	0.194
PFTAIRE1	-0.610	-0.909	0.825	0.281
PCTAIRE2	-0.605	-0.669	0.592	0.397
p70S6K[beta]	-0.921	-0.797	0.331	0.108
mTOR/FRAP	-1.422	-0.713	0.164	0.132
Fyn	-0.060	0.663	-0.198	-0.044
DYRK1A	-0.172	-0.156	0.541	0.000
DAPK3	-0.665	-0.408	0.859	0.352
DAPK1	-0.519	-0.439	0.914	0.489
CK1[epsilon]	-0.556	-0.983	0.581	-0.116
CK1[delta]	-0.442	-0.834	0.533	-0.117
CHK2	-0.779	-0.750	0.461	0.210
CHK1	-0.535	-0.372	0.515	0.456
CDK10	-0.575	-0.894	0.372	0.230
CaMK4	-0.709	-1.007	0.406	0.207
AurA/Aur2	-0.541	-0.721	0.519	0.601
AMPK[alpha]1	-0.893	-0.789	0.452	0.352
AMPK[alpha]1	-0.893	-0.789	0.452	0.352
Akt2/PKB[beta]	-0.770	-0.728	0.351	0.267
Akt1/PKB[alpha]	-0.743	-0.756	0.330	0.263

Table S5. Mean kinase statistic of PKT and STK activation after TGX-221 inhibition. Human PMNs (n=3) were stimulated with ICs in presence of 10 µM TGX-221 for the indicated time points in comparison to IC stimulated PMNs without TGX-221. The mean kinase statistic is shown here as grey if upregulated (positive value) or downregulated (negative value) in comparison to PMNs if they had a mean specificity score of 1 and a significance score of 0.5.

Kinase	0 min	2 min	8 min	15 min
SgK307	-0.847	0.308	0.549	0.012
SGK1	-0.627	0.548	0.954	0.097
RSKL1	-0.741	0.213	0.695	0.390
RSK1/p90RSK	-0.582	0.801	0.198	0.198
PRKY	-0.841	0.305	0.423	0.153
PRKX	-0.726	0.385	0.272	0.221
PKC[theta]	-0.529	0.551	0.386	0.124
PKC[delta]	-0.576	0.627	0.389	0.164
p70S6K[beta]	-0.731	0.484	0.397	0.248
p38[gamma]	-0.811	-0.076	0.490	-0.117
DAPK3	-0.342	0.223	0.852	0.247
DAPK1	-0.542	1.108	0.743	0.107
CHK1	-0.400	0.026	0.563	0.404
CDK5	-0.762	-0.122	0.523	-0.131
AMPK[alpha]1	-0.606	0.555	0.589	0.205
Akt2/PKB[beta]	-0.727	0.510	0.367	0.161
Akt1/PKB[alpha]	-0.713	0.448	0.343	0.232

Table S6. Mean kinase statistic of PKT and STK activation after alpelisib inhibition. Human PMNs (n=3) were stimulated with ICs in presence of 1 µM alpelisib for the indicated time points in comparison to IC stimulated PMNs without TGX-221. The mean kinase statistic is shown here as grey if upregulated (positive value) or downregulated (negative value) in comparison to PMNs if they had a mean specificity score of 1 and a significance score of 0.5.

Kinase	0min	2min	8min	15min
TNIK/ZC2	-0.645	-1.940	0.473	0.374
SGK2	-0.413	-1.413	0.373	0.444
SGK1	-0.468	-2.164	0.956	0.371
RSK3	-0.462	-1.745	0.665	0.611
RSK2	-0.391	-1.496	0.481	0.726
RSK1/p90RSK	-0.375	-1.851	0.736	0.726
RAF1	-0.331	-1.762	0.901	0.487
PRKY	-0.513	-1.682	0.622	0.435
PRKX	-0.457	-1.489	0.594	0.609
PKG2	-0.396	-1.347	0.606	0.648
PKG1	-0.397	-1.363	0.524	0.665
PKD1	-0.319	-1.703	0.664	0.772
PKC[delta]	-0.387	-1.493	0.584	0.649
PKA[alpha]	-0.429	-1.468	0.566	0.603
Pim3	-0.411	-1.307	0.523	0.548
Pim2	-0.395	-1.367	0.590	0.608
Pim1	-0.402	-1.322	0.521	0.554
PFTAIRE2	-0.379	-1.069	0.883	0.455
PFTAIRE1	-0.354	-1.262	1.330	0.681
p70S6K[beta]	-0.421	-1.470	0.582	0.367
p70S6K	-0.421	-1.540	0.582	0.367
mTOR/FRAP	-0.643	-1.722	0.358	0.302
MSK2	-0.454	-1.735	0.332	0.878
MSK1	-0.385	-1.597	0.591	0.684
MAPK14	-0.506	-0.747	0.816	0.157
Lyn	-0.170	-0.297	-0.628	-0.214
HGK/ZC1	-0.481	-1.671	0.824	0.454
DCAMKL1	-0.391	-1.195	0.978	0.687
DAPK3	-0.340	-0.836	1.244	0.820
DAPK1	-0.376	-1.312	1.583	0.590
CK2[alpha]1	-0.422	-1.190	1.592	0.419
CK1[epsilon]	-0.347	-0.819	1.121	0.346

CK1[delta]	-0.316	-0.674	1.250	0.339
CHK2	-0.406	-1.316	0.716	0.632
CHK1	-0.339	-0.898	0.949	0.507
CDKL1	-0.692	-1.068	0.636	0.084
CDK5	-0.459	-0.716	0.844	0.037
CDK11	-0.579	-0.863	0.273	-0.216
AurA/Aur2	-0.414	-1.200	0.405	0.873
ATR	-0.364	-1.012	0.894	0.313
AMPK[alpha]1	-0.497	-1.640	0.888	0.735
Akt2/PKB[beta]	-0.447	-1.668	0.620	0.569
Akt1/PKB[alpha]	-0.440	-1.715	0.632	0.596

Table S7. Mean kinase statistic of PKT and STK activation after AS-604850 inhibition. Human PMNs (n=3) were stimulated with ICs in presence of 5 µM AS-604850 for the indicated time points in comparison to IC stimulated PMNs without AS-604850. The mean kinase statistic is shown here as grey if upregulated (positive value) or downregulated (negative value) in comparison to PMNs if they had a mean specificity score of 1 and a significance score of 0.5.

Supplementary References

Bird, J.E., Smith, P.L., Bostwick, J.S., Shipkova, P., and Schumacher, W.A. (2011). Bleeding response induced by anti-thrombotic doses of a phosphoinositide 3-kinase (PI3K)-beta inhibitor in mice. *Thromb Res* 127(6)**,** 560-564. doi: 10.1016/j.thromres.2011.02.007.

Camps, M., Ruckle, T., Ji, H., Ardissone, V., Rintelen, F., Shaw, J., et al. (2005). Blockade of PI3Kgamma suppresses joint inflammation and damage in mouse models of rheumatoid arthritis. *Nat Med* 11(9)**,** 936-943. doi: 10.1038/nm1284.

Cushing, T.D., Hao, X., Shin, Y., Andrews, K., Brown, M., Cardozo, M., et al. (2015). Discovery and in vivo evaluation of (S)-N-(1-(7-fluoro-2-(pyridin-2-yl)quinolin-3-yl)ethyl)-9H-purin-6-amine (AMG319) and related PI3Kdelta inhibitors for inflammation and autoimmune disease. *J Med Chem* 58(1)**,** 480-511. doi: 10.1021/jm501624r.

Junttila, T.T., Akita, R.W., Parsons, K., Fields, C., Lewis Phillips, G.D., Friedman, L.S., et al. (2009). Ligand-independent HER2/HER3/PI3K complex is disrupted by trastuzumab and is effectively inhibited by the PI3K inhibitor GDC-0941. *Cancer Cell* 15(5)**,** 429-440. doi: 10.1016/j.ccr.2009.03.020.

Son, M.K., Ryu, Y.L., Jung, K.H., Lee, H., Lee, H.S., Yan, H.H., et al. (2013). HS-173, a novel PI3K inhibitor, attenuates the activation of hepatic stellate cells in liver fibrosis. *Sci Rep* 3**,** 3470. doi: 10.1038/srep03470.

Soond, D.R., Bjorgo, E., Moltu, K., Dale, V.Q., Patton, D.T., Torgersen, K.M., et al. (2010). PI3K p110delta regulates T-cell cytokine production during primary and secondary immune responses in mice and humans. *Blood* 115(11)**,** 2203-2213. doi: 10.1182/blood-2009-07-232330.

Yuan, Y., Wen, W., Yost, S.E., Xing, Q., Yan, J., Han, E.S., et al. (2019). Combination therapy with BYL719 and LEE011 is synergistic and causes a greater suppression of p-S6 in triple negative breast cancer. *Sci Rep* 9(1)**,** 7509. doi: 10.1038/s41598-019-43429-7.
